# An inflamed tumor cell subpopulation promotes chemotherapy resistance in triple negative breast cancer

**DOI:** 10.1038/s41598-024-53999-w

**Published:** 2024-02-14

**Authors:** Mauricio Jacobo Jacobo, Hayley J. Donnella, Sushil Sobti, Swati Kaushik, Andrei Goga, Sourav Bandyopadhyay

**Affiliations:** 1 Department of Bioengineering and Therapeutic Sciences, University of California San Francisco, San Francisco, CA 94143 USA; 2 Department of Cell & Tissue Biology, University of California San Francisco, San Francisco, CA 94143 USA; 3 Department of Medicine, University of California San Francisco, San Francisco, CA 94143 USA

**Keywords:** Breast cancer, Tumour heterogeneity, Cancer therapeutic resistance, Chemotherapy

## Abstract

Individual cancers are composed of heterogeneous tumor cells with distinct phenotypes and genotypes, with triple negative breast cancers (TNBC) demonstrating the most heterogeneity among breast cancer types. Variability in transcriptional phenotypes could meaningfully limit the efficacy of monotherapies and fuel drug resistance, although to an unknown extent. To determine if transcriptional differences between tumor cells lead to differential drug responses we performed single cell RNA-seq on cell line and PDX models of breast cancer revealing cell subpopulations in states associated with resistance to standard-of-care therapies. We found that TNBC models contained a subpopulation in an inflamed cellular state, often also present in human breast cancer samples. Inflamed cells display evidence of heightened cGAS/STING signaling which we demonstrate is sufficient to cause tumor cell resistance to chemotherapy. Accordingly, inflamed cells were enriched in human tumors taken after neoadjuvant chemotherapy and associated with early recurrence, highlighting the potential for diverse tumor cell states to promote drug resistance.

## Introduction

Intratumoral heterogeneity (ITH) denotes the presence of cancer cell subpopulations that differ in their genetic, phenotypic or behavioral characteristics imparting the ability to overcome various selective pressures^1,2^. ITH can occur across and within disease sites leading to spatial and longitudinal variation^3,4^ which can promote various aspects of tumor progression and metastasis. ITH is associated with drug resistance and poor outcomes in breast and other cancers^5,6^. As the most aggressive disease subtype accounting for 12 to 18% of all cases and associated with the poorest of outcomes^7,8^, triple-negative breast cancers (TNBCs) display profound ITH^9^. ITH could result in the expansion of resistant cells with genetic or non-genetic differences^3,4^. While genetic differences between cancer cells have been shown to pre-exist^10–12^, much less is known about pre-existing non-genetic variability in cancer cells which could arise from stochastic fluctuations, variation in the tumor microenvironment and spatial localization^13,14^. However, only certain types of non-genetic variability may be relevant to impact clinical outcomes such as drug resistance. Defining axes of variation in cell states and identifying those leading to altered drug responses and outcomes in cancer patients remains a challenge.

Drug resistance is a major concern in breast cancer complicating the treatment of both metastatic disease which is largely incurable and localized disease where there is a 40 to 80% risk of recurrence after neoadjuvant therapy^15–17^. Drug resistance is a ubiquitous problem impacting responses for both chemotherapy as well as targeted therapies. For example, HER2 inhibitor treatment leads to a pathologic complete response in only 25 to 30% of HER2 + breast cancers^18,19^. It has been suggested that ITH is a potential significant factor impeding treatment of breast cancers^20,21^. Although ITH can lead to the Darwinian selection of pre-existing genetically diverse subclones^9,22,23^, clinical evidence from breast cancers undergoing neoadjuvant therapy have indicated treatment often does not result in subclonal selection demonstrating that it is not necessarily genetically encoded^9,24–26^. These data highlight the importance of understanding potential non-genetic mechanisms that contribute to drug resistance, which could illuminate new treatment approaches and regimes.

We hypothesized that transcriptional heterogeneity among individual cells in a tumor sample could encode differences in cell states and associated drug responses. To test this hypothesis we first measured transcript levels in single cells from various breast cancer models, both in vitro using breast cancer cell lines and in vivo using patient-derived xenograft samples, and identified distinct subpopulations in each model tested. To functionalize these data we developed a computational approach based on statistical modeling of the differences observed between individual cancer cell lines with known drug responses that we then used to identify subpopulations with intrinsic resistance to standard-of-care therapies. In TNBC samples we identified a recurrent subpopulation in an inflamed cellular state defined by upregulation of classical interferon stimulated genes (ISGs) which we predicted and validated to be resistant to chemotherapy. Intriguingly, inflamed cells are often enriched in residual tumors after chemotherapy where they are more likely to recur, linking this subpopulation with a clinical need. Here we provide an effective new framework to systematically discover clinically relevant drug resistant cancer subpopulations by predicting drug responses of individual breast cancer cells.

## Results

### Single-cell heterogeneity is pervasive across various models of breast cancer

**Figure 1:**
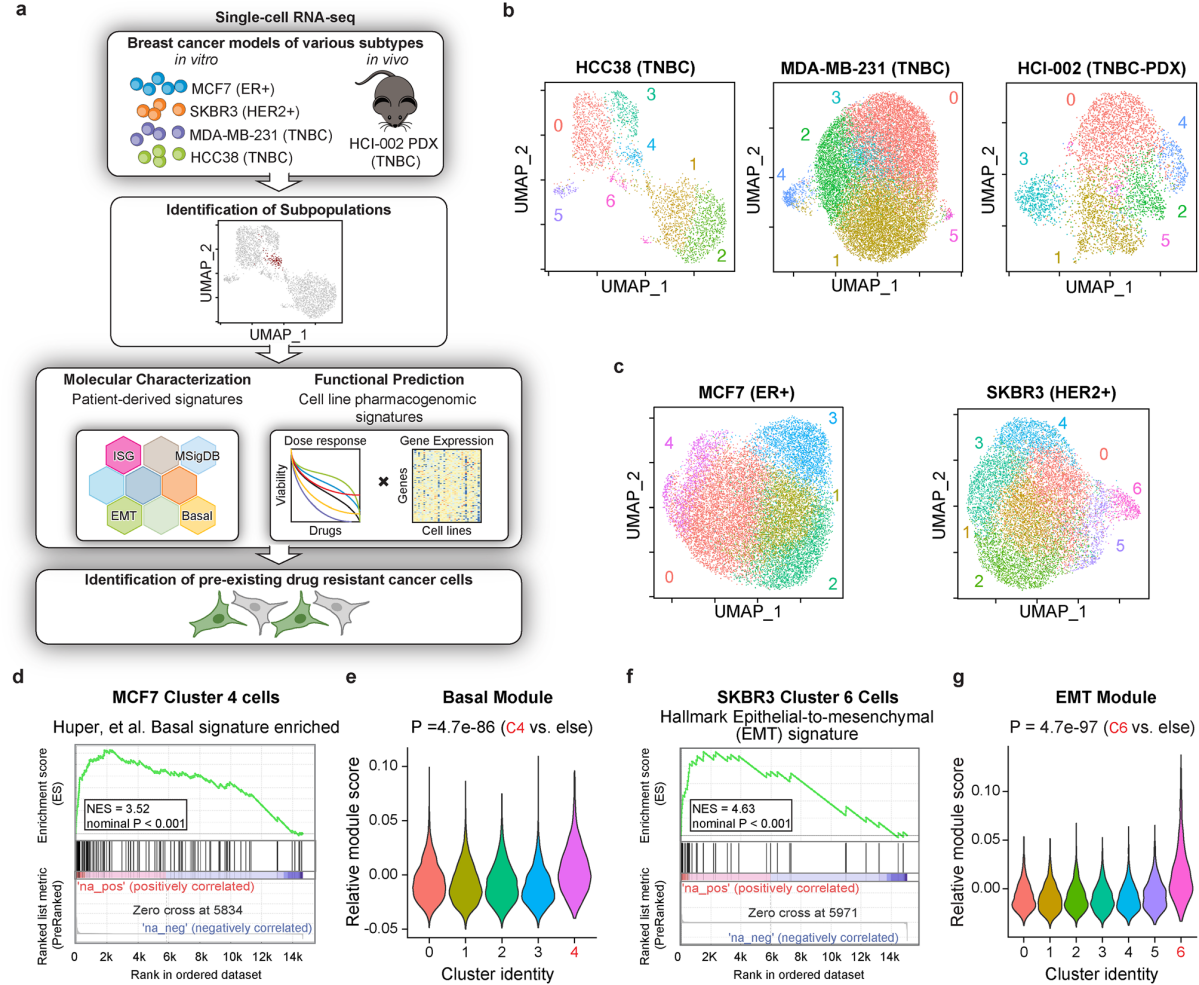
Single-cell RNA-seq identified transcriptional variability in multiple models of breast cancer. (**a**) Schematic for molecular and functional characterization of single-cell RNA-seq data in various model systems. (**b**) UMAP projection of single cell RNAseq data from MDA-MB-231 and HCC38 TNBC breast cancer cell lines and the HCI-002 TNBC-PDX model. Clusters determined by optimized Louvain clustering. (**c**) UMAP projection of scRNA-seq data from the ER + MCF7 cell line and HER2 + SKBR3 cell line with optimized Louvain clustering shown. (**d**) Gene set enrichment analysis (GSEA) of a basal gene signature performed on transcript profiles from MCF7 Cluster 4 cells. NES, normalized enrichment score. (**e**) Relative scores for a basal module in MCF7 clusters. (**f**) Gene set enrichment analysis (GSEA) of a EMT gene signature performed on transcript profiles from SKBR3 Cluster 6 cells. NES, normalized enrichment score. (**g**) Relative scores for an EMT module in SKBR3 clusters. In all graphs* P* values are calculated using a two-sided Wilcoxon test as indicated. *TNBC* triple-negative breast cancer, *PDX* patient-derived xenograft.

To determine the extent of the phenotypic differences between cancer cells in the same sample we performed single-cell transcript profiling on a panel of cell lines representing various subtypes of breast cancer including receptor positive ER + (MCF7), HER2 + (SKBR3) and TNBC (MDA-MB-231, HCC38) cell lines as well as freshly dissociated cells from a patient-derived xenograft (PDX) model of TNBC (HCI-002) using the 10 × Genomics Chromium platform (Fig. 1a). On average per cell we sequenced 23,573 reads, reflecting 6,491 unique mRNA molecules and 1,609 genes after quality control filters. In total after filtering we obtained expression profiles of 54,599 single cells (between 3080 and 19,173 cells per sample) from 5 breast cancer models (Supplementary Datatset 1). To avoid batch effects, all samples were processed together with the exception of HCC38 cells. To categorize phenotypic differences we began by identifying transcriptionally distinct subpopulations present within each sample using a principal component analysis based on the top most significantly variable genes and performed *K*-means clustering on these components to identify the optimal number of distinct subpopulations, as described previously^27^. This approach identified a statistically optimal partition of between 5 and 7 subpopulations within each model (Fig. 1b and c; Supplementary Dataset 2). Taken together, these data uncovers the existence of transcriptionally distinct subpopulations within models of breast cancer thought to be generally homogenous.

### Drug resistance pathways are variably expressed across individual tumor cell supopulations

Within established cell lines we sought to identify clinically relevant subpopulations which may have distinct biological and phenotypic characteristics as a proof of concept. We first performed an unbiased analysis of expression profiles of the 5 subpopulations identified in the ER + luminal MCF7 cell line (Fig. 1c). Cells contained within Cluster 4 made up the smallest fraction of total cells (approximately 7%) and gene set enrichment analysis (GSEA) of differentially expressed genes in this subpopulation showed a significant enrichment for genes upregulated in basal mammary epithelial cells^28^ as well as a basal tumor gene module derived from analysis of variably expressed genes detected across distinct TCGA Breast tumors^29^ (*P* < 0.001, Fig. 1d and e; Supplementary Datset 3). These results indicate the presence of a basal-like subpopulation in a luminal breast cancer cell line that could harbor lower estrogen dependency leading to resistance to hormone therapy^30,31^. Similarly, in HER2-amplified SKBR3 cells we identified a small group of cells (Cluster 6, 4%) that had an enrichment for genes upregulated in the EMT gene signature from MSigDB^32^ and a significantly higher TCGA-derived epithelial-mesenchymal transition (EMT) module score^29^ (*P* < 0.001, Fig. 1c,f,g; Supplementary Dataset 3). EMT is associated with resistance to multiple targeted therapies including HER2 inhibitors^33,34^. These data suggest that some of the variability in gene expression between bulk cancer samples could be due to differences in cell state composition potentially providing insights into the mechanism of emergence of drug resistant cells.

### A subpopulation of cells in an inflamed cellular state is recurrent in TNBCs

**Figure 2:**
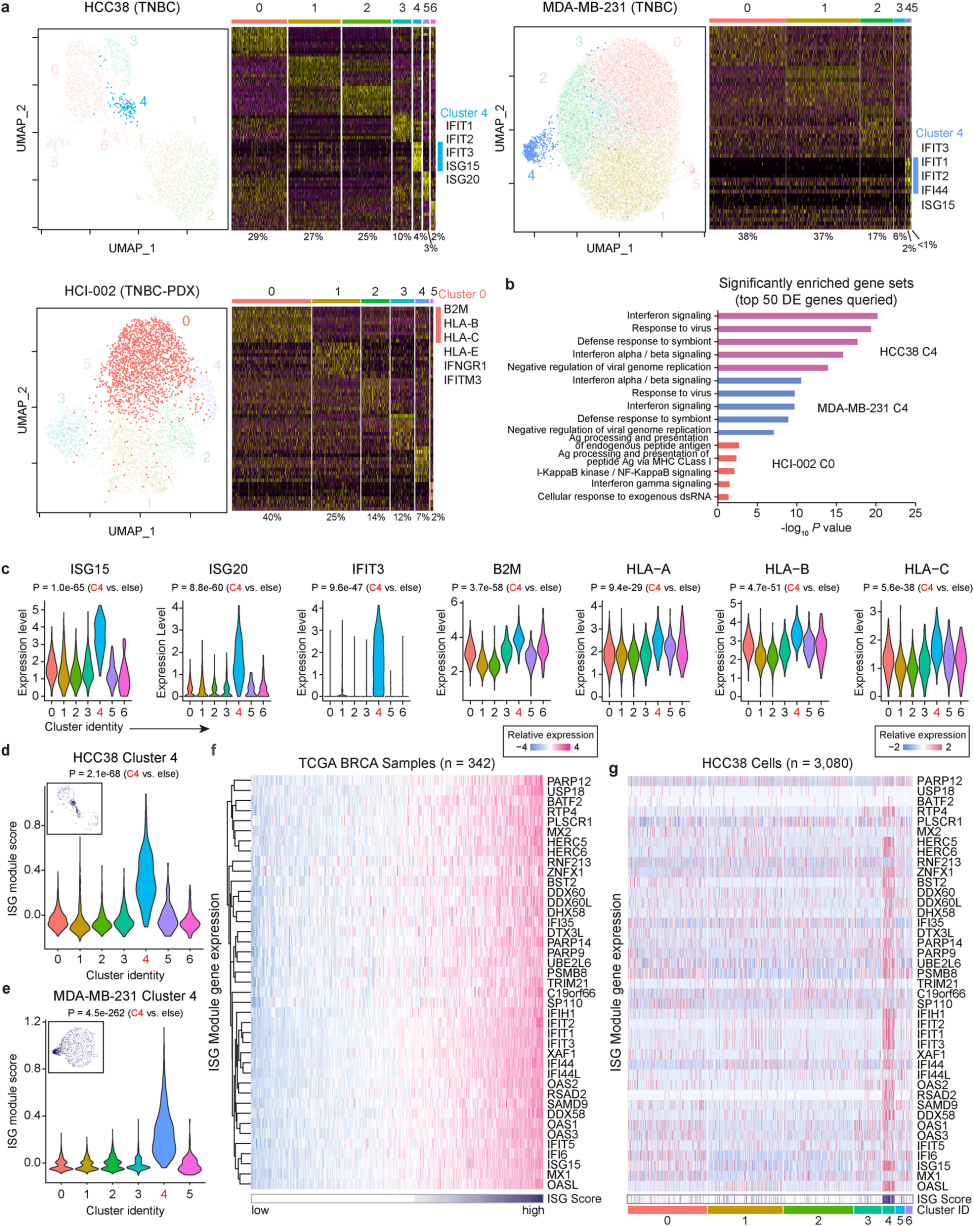
A subpopulation of inflamed cells is recurrent in TNBCs. (**a**) UMAP plots for cells from two TNBC cell lines (HCC38, MDA-MB-231) and a patient-derived xenograft model of TNBC (HCI-002) with optimized Louvain clustering shown and highlighting clusters enriched for ISG genes. Heatmaps displaying scaled expression patterns of top marker genes within each cluster shown to the right with high expression in yellow and low expression in purple. Percentage of total cells contained in each cluster is listed, and inflamed clusters highlighted with accompanying top differentially expressed genes. (**b**) Top significantly enriched gene sets identified from a Gene Set Enrichment Analysis (GSEA) of the 50 most differentially expressed (DE) genes from HCC38 Cluster 4, MDA-MB-231 Cluster 4, and HCI-002 Cluster 0 cells. (**c**) Expression levels of known interferon-stimulated genes for individual HCC38 cells in each cluster. (**d**,**e**) Relative ISG module scores for individual (**d**) HCC38 and (**e**) MDA-MB-231 cells in each cluster. Insert shows ISG module expression for individual cells mapped onto a UMAP plot. (**f**) Relative mRNA expression levels for ISG module genes across the breast cancer TCGA cohort. Samples sorted based on ISG module score. (**g**) Relative expression levels for ISG module genes across individual HCC38 cells. Cells are arranged based on cluster identity and annotated for ISG module score (white indicates low, and purple indicates high). In all graphs* P* values are calculated using a two-sided Wilcoxon test unless indicated otherwise. *ISG* interferon stimulated genes.

Analysis of the gene expression profiles of cells from two TNBC cell lines (HCC38, MDA-MB-231) and a patient-derived xenograft model of TNBC (HCI-002)^35^ identified a subpopulation enriched for genes involved in interferon signaling present in every TNBC model tested which constitute the described inflamed cellular state (HCC38 Cluster 4, 4%; MDA-MB-231 Cluster 4, 2%; HCI-002 Cluster 0, 40%) (Fig. 2a). Analysis of the top 50 differentially expressed genes from each of these subpopulations showed a significant enrichment for interferon signaling and response to virus, including selective enrichment for interferon responsive genes (*ISG15*, *ISG20,* and *IFIT3*) and genes involved in antigen presentation (*HLA-A*, *HLA-B*, and *HLA-C*) in HCC38 Cluster 4 cells (Fig. 2b and 2c, Supplementary Datatset 2). There was strong overlap between differentially expressed genes and independent gene modules based on co-expression across various breast cancer cohorts as well as independent interferon-stimulated gene (ISG) signatures^29,36,37^ (Supplementary Fig. 1a,b). For example, HCC38 Cluster 4 , MDA-MB-231 Cluster 4, and HCI-002 Cluster 0 cells were highly enriched for the tumor cell specific ISG gene signature derived from Liu, et al.^36^ (Supplementary Fig. 1c) as well as a breast cancer specific ISG module composed of 41 genes (Fig. 2d,e, Supplementary Fig. 1d,e, Supplementary Datatset 4). Analysis of the 41 genes across The Cancer Genome Atlas dataset^38^ (TCGA, *n* = 342) showed high variability in gene expression across samples, which was similar to the variability observed between individual HCC38 cells (Fig. 2f,g). While the influence of the immune tumor microenvironment on ISG expression will still need to be established, these data suggest an alternative model whereby differential abundance of cells in an inflamed cellular state could additionally contribute to the variability in ISG expression observed between patients.

To determine if inflamed cells collectively form a genetically defined subclone, we inferred copy number variation (CNV) in each cell using the inferCNV algorithm which estimates CNV levels based on coordinate expression of contiguous genes^39^. Hierarchical clustering identified multiple distinct genetic subclones in HCC38 and MDA-MB-231 cells and inflamed cells were not limited to certain subclones (Supplementary Fig. 2). Although this analysis underestimates the contribution of genetic heterogeneity since it only approximates copy-number changes, it appears that the presence of inflamed cells is not strictly related to genetic subclones.

### Inflamed cells display heightened cGAS-STING pathway activation and genomic instability

**Figure 3:**
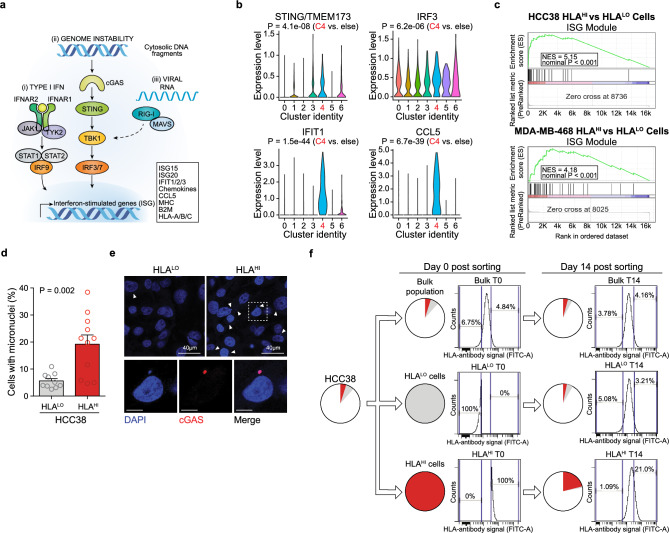
Inflamed cells display heightened cGAS/STING-pathway activation and genomic instability. (**a**) Processes that lead to upregulation of ISG including interferon signaling (IFN), genomic instability channeled through cGAS/STING-pathway activity, and detection of viral RNAs. (**b**) Expression levels of STING and STING effector genes for individual HCC38 cells based on cluster identity. *P* values are calculated using a two-sided Wilcoxon test. (**c**) Gene set enrichment analysis (GSEA) of the ISG module gene signature performed on transcript profiles from HCC38 (top) and MDA-MB-231 (bottom) HLA^HI^ sorted cells. NES, normalized enrichment score. Data representative of *n* = 2 independent experiments. (**d**) Percentage of cells positive for the presence of micronuclei in HCC38 HLA^HI^ (top 5%) and HLA^LO^ (bottom 10%) cells. Data is an average of at least five high-powered (63 ×) fields analyzed per sample (≥ 150 cells/field). Error bars are mean + s.e.m., and *P* value calculated using a two-sided *t*-test. (**e**) Representative images of HCC38 HLA^LO^ (top left) and HLA^HI^ (top right) cells with DAPI (blue) staining DNA. Arrows indicate micronuclei. Higher magnification of HCC38 HLA^HI^ cells positive for micronuclei shown below with co-staining for cGAS (red). Scale bars 10 µm unless indicated otherwise. (**f**) HCC38 cells were sorted into HLA^HI^ (top 5%) and HLA^LO^ (bottom 10%) populations and re-analyzed after 14 d of cell culture. Pie charts depict relative proportions of HLA^HI^ (red) and HLA^LO^ (grey) subpopulations. Data representative of *n* = 2 independent experiments.

We next investigated the mechanism underlying ISG expression in this inflamed subpopulation in TNBCs. Tumor derived ISG expression could stem from response to exogenous interferon or cytosolic nucleic acid sensing due to viral infection or genomic instability, the latter resulting in chronic cGAS-STING pathway activation^36,40^ (Fig. 3a). We detected no expression of interferon genes *IFNA1*, *IFNB1*, or *IFNG* in our TNBC single cell RNAseq data. Given that TNBCs are known to have higher levels of genomic instability^11,38,41–43^, we hypothesized that recurrent inflamed subpopulations may be a result of genomic instability-mediated STING pathway activation^44^. In support of this model, expression of STING (*TMEM173*) and STING effector genes (*IRF3, IFIT1, CCL5*) was elevated in HCC38 Cluster 4 cells (Fig. 3b). We leveraged the fact that a core component of the inflamed program is upregulation of antigen presentation machinery^42^, which was evident in HCC38 Cluster 4 cells (Fig. 2c), and used a pan-HLA (HLA-A/B/C) antibody for isolating and studying inflamed subpopulations in HCC38 and MDA-MB-468 (Supplementary Fig. 3a,b). We found that HLA^HI^ cells had an overall significant enrichment for ISG module genes via RNA-seq and increased abundance of total IFIT1 protein confirming heightened inflammatory signaling (Fig. 3c, Supplmentary Fig. 3c, Supplementary Dataset 5). We observed a significantly higher number of micronuclei in HCC38 HLA^HI^ cells compared to the HLA^LO^ fraction and these micronuclei stained positive for cGAS, whose binding to DNA activates STING through the generation of cyclic GMP-AMP (cGAMP) (Fig. 3d,e). Hence heightened genomic instability triggers cGAS-STING pathway activation leading to the inflamed state. Furthermore, the inflamed state (HLA^HI^) was reversible and dynamically generated from HLA^LO^ cells over the course of two weeks in culture further indicating that this state is not genetically encoded and not a result of long-term chronic pathway activation (Fig. 3f).

### TNBC cells in the inflamed cellular state are chemoresistant

**Figure 4:**
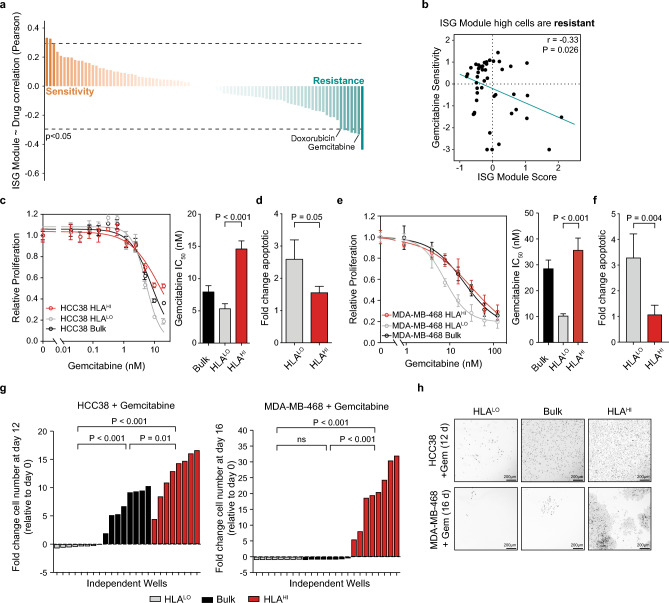
TNBC cells in the inflamed state are chemoresistant. (**a**) ISG module scores from a panel of 84 breast cancer cell lines were correlated with drug sensitivity values across 90 compounds. Sorted Pearson correlation values shown with a cutoff for significant (p = 0.05) correlations indicated by a dashed line. (**b**) Scatter plot of ISG module scores in breast cancer cell lines compared with their sensitivity (normalized –log of IC_50_) to gemcitabine. *P* value based on Pearson correlation. (**c**) Proliferation of HCC38 HLA^HI^ (top 5%), HLA^LO^ (bottom 10%), and bulk population in response to 72 h gemcitabine treatment compared to DMSO control. IC_50_ quantification of dose–response curves shown to the right. (**d**) Fold change in apoptotic cells in HCC38 HLA subpopulations after 72 h treatment with the IC_50_ dose (5 nM) of gemcitabine normalized to DMSO control. (**e**) Proliferation of MDA-MB-468 HLA^HI^, HLA^LO^ and bulk populations in response to 72 h gemcitabine treatment compared to DMSO control. IC_50_ quantification of dose–response curves shown to the right. (**f**) Fold change in apoptotic cells in MDA-MB-468 HLA subpopulations after 72 h treatment with the IC_50_ dose (7.8 nM) of gemcitabine normalized to DMSO control. (**g**) Fold change in the number of cells remaining for HCC38(left) and MDA-MB-468 (right) samples treated for the indicated time period with 2.5 nM or 11 nM gemcitabine relative to day 0 calculated for *n* = 8 independent samples. For comparison, the mean day 0 cell count from *n* = 4 independent samples was used. (**h**) Representative images of colony formation assay for HCC38 (top) and MDA-MB-468 (top) HLA^LO^ (left), bulk (middle), and HLA^HI^ (right) cells following chemotherapy treatment. Cells were stained with Hoechst 33,342 and fluorescent image inverted for clarity. Scale bar, 200 µm. For (**c**–**f**), data represents *n* = 4 biologically independent samples. Error bars are mean ± s.d., and *P* values calculated using a two-sided *t*-test except for (**g**) in which a two-sided *t*-test with Welch’s correction was used.

Given their association with genomic instability and distinctive upregulation of ISGs apart from the bulk population, we investigated the functional consequence inflamed cells may have on therapeutic responses. We performed pharmacogenomic modeling based on bulk breast cancer cell line drug sensitivities using a panel of 84 molecularly characterized breast cancer cell lines^45^ where the ISG module score was calculated from bulk RNA-seq data. Drug response data (IC_50_) for 90 compounds measured across these cell lines was individually correlated with baseline ISG module score to identify compounds whose efficacy was linked with cells in an inflamed state (Fig. 4a). Cell lines with higher ISG module scores were significantly associated with resistance to the chemotherapy agent gemcitabine (r = -0.33, *P* = 0.026) (Fig. 4b). To test if inflamed cells were resistant to gemcitabine we flow sorted HCC38 and MDA-MB-468 HLA^HI^ and HLA^LO^ subpopulations, confirmed that they proliferate at approximately equal rates, and tested their response to gemcitabine in a competition assay (Supplementary Fig. 4a,b). As predicted by the model, HCC38 and MDA-MB-468 HLA^HI^ cells were more resistant to gemcitabine compared to HLA^LO^ cells as evidenced by their increased proliferation and decreased apoptosis following a 72 h drug treatment at multiple doses (Fig. 4c-f).

Since inflamed cells displayed intrinsic chemotherapy resistance, we hypothesized that this population may also contribute to residual disease after high dose chemotherapy, thereby fueling acquired restance. To test whether pre-existing HCC38 and MDA-MB-468 inflamed cells cause long term resistance to chemotherapy HLA sorted cells were treated for approximately two weeks with a high dose (IC_80_) of gemcitabine and final cell numbers compared relative to the initial cell numbers. Even in the presence of such a high dose of gemcitabine HCC38 HLA^HI^ cells grew more robustly after treatment (12.3-fold more cells than initially seed) compared to HLA^LO^ and bulk cells (-0.44 fold and 7.17-fold, respectively) (Fig. 4g,h). Similarly, MDA-MB-468 HLA^HI^ cells produced 19.87-fold more cells than were initially seeded, whereas HLA^LO^ and bulk cell populations retracted after treatment (-0.97 and -0.85 fold, respectively) (Fig. 4g,h). These data indicate that HLA^HI^ cells are sufficient to generate resistance, and in the case of one of the cell lines, they are necessary for chemotherapy resistance to occur. Hence, tumor cells which pre-exist in an inflammatory state may contribute to disease persistence and eventually cause tumor regrowth and relapse in TNBC.

**Figure 5:**
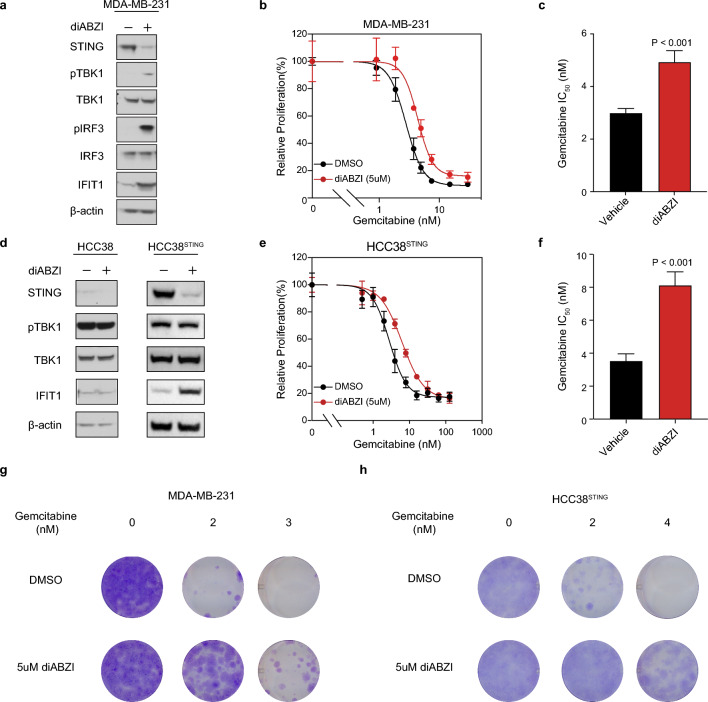
STING activity is sufficient for chemoresistance and contributes to drug tolerance and acquired resistance in vitro. (**a**) Immunoblot of lysates taken after 24 h of 5uM diABZI or DMSO treatment in MDA-MB-231 cells with the indicated antibodies. β-actin is shown as a loading control. Representative image from *n* = 3 independent experiments. (**b**) Proliferation of MDA-MB-231 cells in response to 24 h diABZI or DMSO pre-treatment followed by 72 h gemcitabine co-treatment. (**c**) IC_50_ quantification of MDA-MB-231 dose–response curve (**b**). (**d**) Immunoblot of lysates taken after 24 h of 5uM diABZI or DMSO treatment in HCC38 (left) and HCC38^STING^ overexpressing cells (right) with the indicated antibodies. β-actin is shown as a loading control. Representative image from *n* = 3 independent experiments. (**e**) Proliferation of HCC38^STING^ overexpressing cells in response to 24 h diABZI or DMSO pre-treatment followed by 72 h gemcitabine co-treatment. (**f**) IC_50_ quantification of HCC38^STING^ dose–response curve (**e**). (**g,h**) Crystal violet staining of MDA-MB-231 (**g**) and HCC38^STING^ overexpressing cells (**h**) after 9 d treatment with increasing concentrations of gemcitabine. Images are representative of *n* = 3 independent experiments with similar results. For (**b**,**c**,**e**,**f**), data represents *n* = 4 biologically independent samples. Error bars are mean ± s.d., and *P* values calculated using a two-sided *t*-test.

To determine whether an inflammatory response resulting from cGAS-STING signaling itself was sufficient to cause resistance to chemotherapy, we used a chemical mimic of the STING ligand, cyclic guanosine monophosphate-adenosine monophosphate (cGAMP), dimeric aminobenzimidazolec (diABZI) which functions as a STING agonist^46^ to activate the pathway in various breast cancer cell lines. Following treatment with diABZI, MDA-MB-231 cells showed heightened phosphorylated TBK1, phosphorylated IRF3, and total IFIT1 protein, confirming robust activation of the cGAS-STING pathway leading to the presence of an inflamed cellular state (Fig. 5a) and consequently resistance to gemcitabine (Fig. 5b,c, Supplementary Fig. 4c). In HCC38 cells, diABZI did not activate signaling, which we assumed to be due to the lack of detectable STING expression in the bulk population (Fig. 5d). Therefore, we reconstituted the pathway by exogenous expression of STING (HCC38^STING^). When treated with diABZI, HCC38^STING^ cells exhibited an increase in IFIT1 protein abundance and a decrease in STING protein abundance similar to that in MDA-MB-231 cells confirming establishment of the inflammatory state (Fig. 5d). HCC38^STING^ cells pretreated with diABZI were more resistant in short-term culture to gemcitabine compared to cells without pretreatment (Fig. 5e,f). Growth rate inhibition analysis^47,48^, which normalizes drug sensitivity by cell division, further confirmed the increased chemoresistance exhibited by diABZI pretreated cells to be independent of differences in growth rates (Supplementary Fig. 4d,e). Moreover, cotreatment of diABZI with gemcitabine demonstrated improved longterm cell survival in both MDA-MB-231 and HCC38^STING^ cells (Fig. 5g,h). Lastly, MDA-MB-468 cells treated with diABZI also demonstrated strong cGAS-STING activation (Supplementary Fig. 4f) resulting an an increased resistance to gemcitabine (Supplementary Fig. 4g). Altogether, these results indicate that STING activation is sufficient to cause resistance to gemcitabine.

### Inflamed cells are enriched in chemotherapy-induced residual disease and associated with poor outcome in TNBC

**Figure 6:**
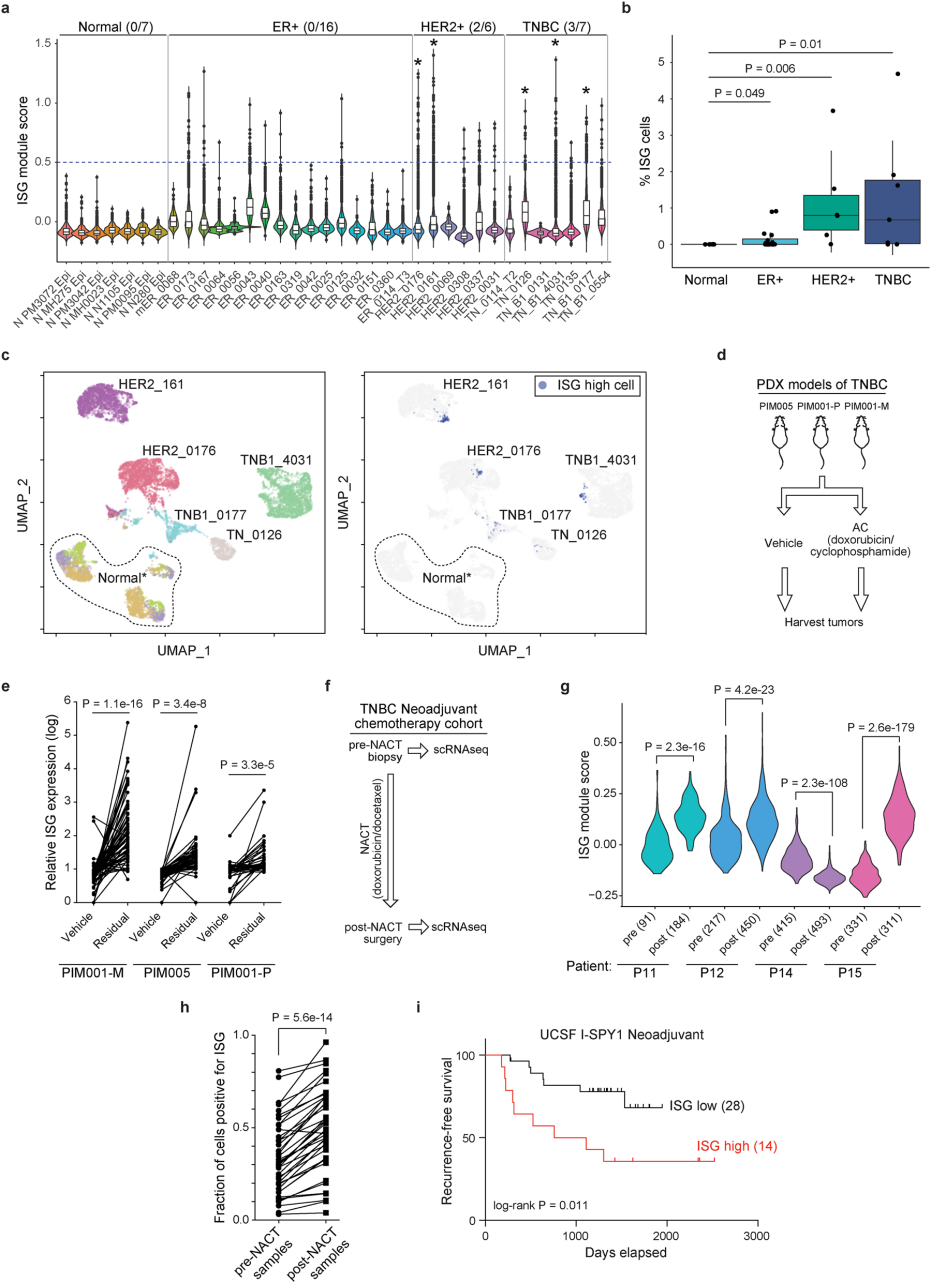
Inflamed cells are enriched in chemotherapy-induced residual disease and associated with poor outcome in TNBC. (**a**) Relative ISG module scores in treatment naïve patient tumors and normal breast tissue samples^49^. Cutoff for cells exhibiting heightened inflammatory signaling is indicated by a dashed line. Number of tumor samples with > 1% inflamed cells is shown per subtype. (**b**) Percentage of inflamed cells in each tumor sample grouped by subtype. Box plots show median, upper/lower quartiles and range from 25–75 percentiles. (**c**) UMAP plot of samples with abundant inflammatory cells colored according to patient identify (left) and ISG cell classification (right). Normal* indicates three normal samples (N MH0023, N N1105, N MH275). (**d**) TNBC PDX models from 3 primary breast tumors were treated with vehicle or a single dose of AC treatment (doxorubicin/cyclophosphamide) and harvested approximately 20 days later for RNA-seq. (**e**) Scatter plot showing relative expression levels for each of the 41 ISG genes in the indicated vehicle and residual tumors following AC treatment. (**f**) Single-cell transcriptome profiles were derived from matched pre- and post-NACT (neoadjuvant chemotherapy containing doxorubicin and docetaxel) samples from 4 TNBC patients^9^. (**g**) Violin plot of relative ISG module scores for pre- and post-NACT cells from each patient shown with number of cells analyzed in each sample indicated. (**h**) Fraction of cells expressing each individual ISG module gene where each point represents a single ISG averaged over 4 matched pre- or post-NACT biopsies. (**i)**The top third of patients whose tumor ISG score was the most elevated in the surgical post-chemotherapy sample compared to pre-treatment were classified as ISG high and the remaining two-thirds as ISG low in the ISPY cohort^51^. Probability of recurrence-free survival (%) is shown and the number of patients in each group indicated. *P* values calculated using a two-sided *t*-test except for (**b**) in which a two-sided Wilcoxon test was used.

We next sought to clarify the role of this subpopulation on chemoresistance in human breast cancers. Our data indicate that inflamed cells are a rare, preexisting subpopulation in TNBC. To determine whether inflamed cells are also present in treatment naïve tumors as a rare population, we analyzed single cell RNA-seq data of breast cancer tissue collected from twenty-nine therapy naïve patients of various breast cancer subtypes and seven normal breast tissue samples^49^ (Fig. 6a). We observed lower expression of the ISG gene signature in normal breast tissue versus tumor samples and a cutoff that excluded normal cells was used to call individual inflamed cells in tumor samples (Fig. 6a). Among the different breast cancer subtypes, inflamed cells were more abundant in TNBC and HER2 + tumors than in ER + tumors (Fig. 6b). We identified five tumors where inflamed cells made up more than 1% of the total, of which two were HER2 + and three TNBC (Fig. 6a, starred). We used a UMAP projection to visualize transcriptional similarities between tumor cells and inflamed cells. While each tumor segregated independently, inflamed cells within three of the five samples clustered together indicating that they form a transcriptionally distinct subpopulation (Fig. 6c). Identification of subclones using the inferCNV algorithm showed minimal genetic similarity among inflamed tumor cells suggesting that this non-genetic, inflamed tumor population is recapitulated within human breast cancers (Supplementary Fig. 5).

Our data also indicate that inflamed cells are more resistant to chemotherapy treatment (Figs. 4, 5). We reasoned that we could glean the most information on the relevance of this population in the setting of neoadjuvant chemotherapy where pre- and post-therapy tissues are collected. Initially using PDX models we analyzed residual disease samples in three TNBC PDX models collected approximately 20 days after a single dose of doxorubicin and cyclophosphamide was used to eliminate the majority of the tumor mass (clinically referred to as Adriamycin/Cytoxan or AC treatment)^50^ (Fig. 6d). Using RNA-seq data from the residual disease samples, all three models displayed enrichment of expression of genes in the ISG module compared to vehicle controls indicating an association between ISG expression and a chemotherapy resistant state (Fig. 6e).

We next evaluated if we could find supporting evidence for the selection of inflamed cells using single-cell RNA-seq data from patients undergoing neoadjuvant chemotherapy treatment (NACT). We processed data from four TNBC patients who had been treated with doxorubicin containing chemotherapy who had single-cell RNA-seq performed on matched tumor samples taken pre- and post-treatment at the time of surgery^9^ (Fig. 6f). The average ISG module score per cell was significantly higher in the post-treatment samples than pre-treatment in 3 out of the 4 patients (P11, P12, P15) (Fig. 6g). For each gene in the ISG module we determined the fraction of cells within a sample which had a non-zero expression and found that there was an overall increase in the percentage of cells positive for any ISG post-NACT, largely driven by 3 out the 4 patients (Fig. 6h; Supplementary Fig. 6a,b). Hence, NACT often results in the enrichment of ISG expressing inflamed tumor cells because they are chemoresistant.

To determine if inflamed tumor cells are linked to eventual relapse, we examined a cohort of breast cancer patients undergoing NACT including doxorubicin from the I-SPY1 trial^51^. Analysis of microarray data from 42 matched pre- and post-NACT breast tumor biopsies revealed that elevated ISG expression in the residual tumor at the time of surgical resection was significantly associated with early recurrence (*P* = 0.011; Fig. 6i; Supplementary Fig. 6c). Many of the individual genes in this module as well as canonical ISGs (e.g. *IFIH1*, *OAS2*, *DDX58*) were significantly elevated in residual tumors that recurred over the course of the study (Supplementary Fig. 6d). Together, these data indicate that chemotherapy often results in the enrichment of tumor cells in the inflamed state where it is associated with early recurrence and disease progression. These data illustrate the utility of this pharmacogenomic approach in predicting responses of single tumor cells and indicate that targeting TNBC cells in an inflamed state may increase the efficacy of chemotherapy regimens by eradicating a resistant subpopulation.

## Discussion

Most cancers harbor high levels of intra-tumor heterogeneity evident by inference of mutant subclones from bulk sequencing data^6,11,12^ as well as single-cell analysis^52–54^. Such clinical observations highlight the importance of understanding functional differences between cells, how such differences arise, and its role on drug resistance with the goal of ultimately designing new therapeutic interventions. To identify if there were differences in tumor cell states in experimentally tractable models, we performed single-cell RNA-seq on breast cancer cell lines and PDX models revealing significant heterogeneity reflected in pathway-specific differences between subpopulations. Although the reconstruction of CNV profiles can only give an approximation of subclonal diversity, our data suggest that transcriptionally distinct subpopulations are usually not also genetically distinct. These data are consistent with previous reports indicating that some but not all variability between cancer cells can be attributed to subclonality^9,55^. Of note, most functionally distinct subpopulations of cells in this study are relatively rare (approximately 5%) and their identification was only possible because we sequenced over 3,000 cells per sample. Lower cell numbers may be a limitation of other single-cell RNA-seq data in breast cancer^9,55,56^. Our data uncover unexpected heterogeneity in cell lines often presumed to be homogenous and suggest that non-genetic factors underlie the majority of differences in cancer cell states.

We identified a rare but recurrent subpopulation of cells in an inflammatory state that was specific to TNBC in vitro and displayed heightened cGAS/STING-pathway activation associated with genomic instability. We identified a similarly distinct tumor subpopulation in at least three TNBC and HER2 + breast cancer samples, raising the possibility that this population is prevalent in human cancers, which will require further exploration.

Through pharmacogenomic modeling and experimental validation, we determined that these inflamed cells are resistant to standard-of-care chemotherapies, in line with previous studies linking ISG activation and resistance to DNA damaging agents^50,57–59^. We found that increasing inflammatory signaling by triggering cGAS-STING activation is sufficient to cause resistance to gemcitabine suggesting the further importance of the cGAS-STING pathway in drug resistance. We present data supporting that this population causes eventual acquired resitance in vitro and chemoresistance in the clinical setting since we observed an increase in the proportion of inflamed cells in patient residual disease samples after chemotherapy and this enrichment is strongly associated with early recurrence. More single cell data from patients will be required to fully characterize the significance of inflammatory subpopulations on patient time to relapse. Mechanisms for the maintenance of inflammatory cells will need to be elucidated. For example, the cause of genomic instability causing STING activation remains unknown. In patients, tumor cell inflammatory signaling should be immunogenic due to increased antigen presentation, and therefore inflamed cells may only exist in a cold immune tumor micro-environment where they are resistant to immunotherapies. However, the identified association between high ISG expression and resistance to immunotherapy^60^ further suggests the inflammatory cellular state may also express and secrete immunosuppressive factors which promote immune evasion in different cancer settings. Beyond their significance in drug resistance, inflammatory cells may also be prone to metastasis, which has been reported to be dependent on STING^61^. Future work could also identify selective vulnerabilities in the inflamed state that could be used to reverse or selectively eliminate this population. This may potentially be accomplished through loss of ISG negative regulators causing aberrant dsRNA accumulation and activation of the double-stranded RNA sensor, protein kinase R (PKR), leading to cell lethality^36^. This study highlights the importance of tumor single cell analysis to define key axes of transcriptional variability that can lead to distinct treatment outcomes and ultimately new treatment strategies tailored by a finer dissection of tumor cell composition.

## Methods

### Breast cancer cell lines and reagents

HCC38, MCF7, and MDA-MB-231 cells were obtained from the American Type Culture Collection (ATCC). SKBR3 and MDA-MB-468 cells were purchased from the UCSF Cell Culture Facility. Cell lines were grown according to published guidelines^62^ except for SKBR3 cells which were cultured using RPMI media supplemented with 10% fetal bovine serum (FBS) and 1% pen/strep. All cell lines tested negative for mycoplasma contamination. All drugs used in this study were purchased from Selleck Chemicals (Gemcitabine and diABZI). To exogenously overexpress *TMEM173* or *STING1*, a *STING1* ORF was cloned into the lentiviral pLX304-Blast-V5 vector as part of the CCSB-Broad Lentiviral Expression Library^63^. The vector was transduced using a lentiviral system and cells were selected using 7.5 *u*g mL^-1^ blasticidin to establish stable HCC38 STING overexpressing cells.

### Tissue dissociation

All protocols described in this section regarding mouse studies wre approved by the UCSF Institutional Animal Care and Use Committee, and all relevant ethical regulations were followed. HCI-002 patient-dervied xenograft (PDX) tumor tissue grown as previously described was a gift from A. Goga^35,64^. HCI-002 PDX tissue was harvested and processed into single-cell suspensions following established protocols^65^. Briefly, PDX tissue was mechanically chopped with scalpels and placed in culture medium DMEM/F12 with 5% FBS, 5 µg ml^−1^ insulin (UCSF Cell Culture Facility), 50 ng ml^−1^ gentamycin (UCSF Cell Culture Facility) and supplemented with 2 mg ml^−1^ collagenase-1 (Sigma). Sample was then digested for 45 min at 37 °C. The resulting suspensions were resuspended in 2 U µl^−1^ DNase (D4263-5VL, Sigma Aldrich) for 3 min at room temperature, washed and dissociated with 2 ml of 0.05% trypsin/EDTA (25–052-CI, Corning) for 10 min at 37 °C. Cell suspensions were then filtered through a 70 µm filter, and frozen in DMEM/F12 with 50% serum, 10% DMSO, and stored in liquid nitrogen prior to study.

### Single-cell and bulk RNA-seq sample preparation and sequencing

Breast cancer cell lines were thawed and carried according to published culture methods as described. Viably frozen PDX cell suspensions were thawed, washed and stained for fluorescence-activated cell sorting (FACS) using fluorescently labeled antibodies for human antigen CD298 (PE; 341704, BioLegend) and mouse antigens CD45 (APC; 559864, BD Pharmingen), CD31 (APC; 551262, BD Pharmingen) and Ter119 (APC; 557909, BD Pharmingen). Flow sorting was done using a BD FACSAria II cell sorter (Becton Dickinson) where contaminating hematopoietic and endothelial cells were excluded by gating out Lin^+^ (CD45, Ter119, CD31) cells. Dead populations from both PDX samples and cell lines were eliminated by excluding Sytox positive (SYTOX Blue Dead Cell Stain, S34857, Life Technologies) cells with cells showing at least 80% viability proceeded with for single-cell sequencing. Sorted cells were washed in PBS with 0.04% BSA and resuspended at a concentration of ~ 1000 cells/µl. Single-cell RNA sequencing was performed at the IHG Genomics Core (UCSF) using the Chromium Single Cell 3ʹ Reagents Kit (CG00026 Rev B., 10 × Genomics), and libraries were prepared following the manufacturer’s protocol. Libraries were then sequenced using the Illumina HiSeq2500 platform to achieve an average depth of 50,000 reads per cell. Single-cell data for the HCC38 cell line was provided by 10 × Genomics.

For bulk RNA seq total RNA was isolated from FACS sorted cells using the RNAeasy Mini kit (Qiagen). RNA was quantified using a Qubit RNA BR (Broad-Range) Assay kit while RNA integrity was determined by the Agilent 2100 Bioanalyzer system. A complementary DNA library was prepared and Illumina RNAseq performed by Novogene (https://www.novogene.com/us-en/). All gene expression analyses were performed using the DESeq2Rpackage version 1.20.0.

### Single-cell RNA-seq data processing and analysis

The Cell Ranger Single-Cell Software Suite version 1.1.0 was used to perform sample demultiplexing, barcode processing and single-cell 3′ gene counting. Clusters were identified in each independent scRNA-seq dataset following the Seurat version 4.3.0 (http://satijalab.org/seurat/) pipeline^66–68^. Initial quality control filtering trimmed the datasets to where each gene was expressed in at least three cells and each cell had at least 200 expressed genes. Cells with greater than 2,500 genes (8,000 genes in the case of HCC38) were further excluded to omit outliers. The percentage of UMIs mapped to mitochondria was set to less than 5%. We identified the top 2,000 variable features using the “vst” method for each sample independently. Cell cycle differences and the number of UMIs in cells were regressed out using the ScaleData function. Principal component analysis (PCA) was then performed using the highly variable genes to reduce dimensionality for each sample individually. Significant principal components were then determined by Jackstraw method and used to perform density clustering to identify the optimal number of clusters in the data, which were then visualized using uniform manifold approximation and projection (UMAP) dimensionality reduction. Differentially expressed genes for each respective cluster were identified using the FindAllMarkers (or FindMarkers) function which ran Wilcoxon rank sum tests.

Molecular programs describing breast cancer biology and representing breast cancer patient variability were previously defined using MAGNETIC^29^. To compare a module across subpopulations, gene module scores were calculated for each individual cell by summing up all genes in a module. This sum was compared to a control gene-set as a normalization factor as described previously^54^. Gene set enrichment analysis (GSEA) of hallmark cancer gene signatures in the Molecular Signatures Database version 2023.1.Hs and MAGNETIC modules was performed using GSEA version 4.3.2 software^28^. Cells belonging to subpopulations identified in the single-cell RNA sequencing dataset were averaged to serve as a representation of each subgroup. Differential expression analysis performed between inflamed and noninflamed cells was used to generate a list of ranked genes based on a score calculated as -log10 of *P* value multiplied by sign of the log2 fold-change value. The minimum and maximum criteria for selection of gene sets from the collection were 15 and 500 genes, respectively. Similarly, pathway over-representation analysis was performed by “clusterProfiler”^69^. Gene sets were considered significantly enriched following a nominal *P* < 0.05 and FDR < 0.25 cutoff.

### CNV estimation based on single-cell RNA-seq data

Large-scale copy number variations (CNVs) were inferred from single-cell expression data using inferCNV version 1.11.2^39^. Initial CNVs were estimated by sorting the analyzed genes by their chromosomal location and applying a moving average to the relative expression values, with a sliding window of 100 genes within each chromosome, as previously described^70^. Hierarchical clustering of CNV profiles was performed and profiles were visualized via heatmap using the InferCNV package’s defaults parameters.

### Fluorescence-activated cell sorting (FACS)

Cancer cells were flow sorted on a Sony SH800S Cell Sorter (Sony) using anti-human HLA-A/B/C conjugated to Alexa Fluor 488 (560169, BD Pharmingen) at the manufacturer’s recommended concentration. Gating of positive and negative cells was defined by the unstained control, and cells were sorted into representative high and low expressing populations as indicated. All sorting was performed to separate a high and low fraction constituting the top 5% and bottom 10% of cells. For subpopulation kinetic experiments, HLA subpopulations were sorted and reanalyzed by FACS immediately post-sorting and at 14 d afterwards. FACS data were analyzed using FlowJo Software version 10.6.1 (Tree Star).

### Immunofluorescence and micronuclei quantification

Cells were seeded on glass coverslips and fixed with 4% paraformaldehyde (PFA) in PBS for 10 min at room temperature. Cells were then permeabilized in 1X PBS/0.3% Triton X-100 for 10 min at room temperature before blocking for 40 min with 3% BSA in PBS. Coverslips were then incubated with primary antibody overnight at 4 °C, followed by incubation with a secondary antibody for 1 h at room temperature. Both primary and secondary antibodies were diluted in blocking buffer given the following dilutions: cGAS (D1D3G) (15102, Cell Signaling) at 1:1,000, anti-rabbit-Alexa 647 (A212245) (Thermo Fisher) at 1:1,000. Coverslips were then mounted using Vectashield Antifade Mounting Medium with DAPI (Vector Laboratories) and imaged using a Zeiss LSM 780 confocal microscope equipped with 25 × and 63 × water immersion objectives and a CCD camera. The images were further processed in ImageJ^71^ and scoring was performed under blinded conditions. Micronuclei positive fractions were calculated as a percentage of total cells per field. For quantification, multiple (3–5) random fields were captured and 700–1000 cells were counted in each independent experiment. Micronuclei were defined as discrete DNA aggregates separate from the primary nucleus in cells where interphase primary nuclear morphology was normal. Cells displaying mitotic morphology and/or with an apoptotic appearance were excluded.

### Drug sensitivity studies

HLA-sorted or bulk cells were seeded in 384-well assay microplates at a density of 1,000 cells/well and allowed to adhere overnight. Following a 72 h drug exposure, proliferation and cell death were measured by staining with Hoechst 33,342 (Thermo Fisher Scientific) nuclear dye and YO-PRO1 (Thermo Fisher Scientific) early apoptosis dye, respectively. Cells in stained plates were analyzed and nuclei counted using a CellInsight High Content microscope (Thermo Fisher Scientific). If necessary, drugs were repleneshid every 4 days. For colony outgrowth assays, cells were seeded in 12-well microplates at a density of 500–3000 cells/well, allowed to adhere overnight, treated with diABZI STING agonist or DMSO vehicle for 24 h, and then exposed to drug or DMSO vehicle for 9 d with medium change and drug refresh every 4 d. Cells were fixed with 100% methanol, stained with 0.5% crystal violet, and imaged using an EPSON Perfection V600 scanner prior to quantification.

### Immunoblotting

Uncropped blots are provided (Supplementary Fig. 7). Cells for immunoblots were collected and lysed using RIPA buffer (50 mM Tris–HCl pH 7.5, 150 mM NaCl, 0.5% sodium deoxycholate, 0.1% SDS, 1 mM EDTA, 1 mM EGTA, 1% NP-40) supplemented with protease and phosphatase inhibitor cocktails (Sigma-Aldrich) for 15 min on ice. Cell lysates were cleared by centrifugation at 14,000 r.p.m. for 10 min at 4 °C. Supernatant was collected and protein quantified by BCA. Equal amounts of protein samples were resolved using 4–12% SDS-PAGE gels (Life Technologies) and transferred to polyvinylidene difluoride membranes (Millipore). Membranes were probed overnight on a 4 °C shaker with primary antibodies (1:1,000 dilution unless indicated) recognizing the following proteins: p-TBK1 (Ser172) (5483, Cell Signaling), TBK1 (3504, Cell Signaling), p-IRF3 (Ser386) (37829, Cell Signaling), IRF3 (11904, Cell Signaling), STING (13647, Cell signaling), IFIT1 (14769, Cell Signaling), and β-actin (3700, Cell Signaling, 1:10,000). Membranes were then incubated with horseradish peroxidase-conjugated secondary antibodies (1: 5,000 dilution) for 1 h at room temperature and developed using an enhanced chemiluminescence solution.

### Analysis of public datasets

We used patient Agilent G4502A_07_3 array gene expression data from the TCGA breast cancer study (BRCA)^38^. Breast cancer cell line drug sensitivities were obtained from Daemen et al.^45^ in which we filtered cell lines not included in drug analysis and missing sensitivity data for more than half of the drugs analyzed. Corresponding Affymetrix GeneChip Human Gene 1.0 ST exon array gene expression data was downloaded from Synapse (https://www.synapse.org/—!Synapse:syn2346643)**.** Treatment naïve breast tumor and normal tissue single cell RNA-seq data previously analyzed and published by Pal et al.^49^ was downloaded from the Gene Expression Omnibus (GEO): GSE161529 and filtered for tumor cells as described in the original publication^49^. AC treated PDX RNA-seq data was obtained from Echeverria, et al^50^. Single cell RNA-seq data from patients undergoing neoadjuvant chemotherapy was in the form of transcripts per million reads (TPM) for each gene per cell and provided by Nicholas Navin^9^. A gene was determined to be expressed in a cell if its TPM > 0. Array-based gene expression data from patients on the I-SPY1 clinical trial^51^ was downloaded from the Gene Expression Omnibus: GSE32603.

### Statistical analysis

Data are expressed as means ± s.d., unless otherwise indicated. Statistical analyses were performed using GraphPad Prism 10 version 10.0.2 and R version 4.1.3. Two-tailed Student *t*-tests were used in all comparisons unless otherwise noted with *P* < 0.05 considered statistically significant throughout the study.

### Supplementary Information

Supplementary Information 1.

Supplementary Information 2.

Supplementary Information 3.

Supplementary Information 4.

Supplementary Information 5.

Supplementary Legends.

Supplementary Figures.

## Data Availability

All data generated or analyzed during this study are included in this published articles and its supplementary information files. Single cell and bulk RNA-seq data is deposited in the NCBI GEO database under GSE250158. Cell lines generated in this study are available upon reasonable request from the authors.

## References

[1] Alizadeh AA (2015). Toward understanding and exploiting tumor heterogeneity. Nat. Med..

[2] Martelotto LG, Ng CK, Piscuoglio S, Weigelt B, Reis-Filho JS (2014). Breast cancer intra-tumor heterogeneity. Breast Cancer Res..

[3] Vitale I, Shema E, Loi S, Galluzzi L (2021). Intratumoral heterogeneity in cancer progression and response to immunotherapy. Nat. Med..

[4] Dagogo-Jack I, Shaw AT (2018). Tumour heterogeneity and resistance to cancer therapies. Nat. Rev. Clin. Oncol..

[5] Morris LGT (2016). Pan-cancer analysis of intratumor heterogeneity as a prognostic determinant of survival. Oncotarget.

[6] Andor N (2016). Pan-cancer analysis of the extent and consequences of intratumor heterogeneity. Nat. Med..

[7] Foulkes WD, Smith IE, Reis-Filho JS (2010). Triple-negative breast cancer. N. Engl. J. Med..

[8] Liedtke C (2008). Response to neoadjuvant therapy and long-term survival in patients with triple-negative breast cancer. J. Clin. Oncol. Off. J. Am. Soc. Clin. Oncol..

[9] Kim C (2018). Chemoresistance evolution in triple-negative breast cancer delineated by single-cell sequencing. Cell.

[10] Navin N (2011). Tumour evolution inferred by single-cell sequencing. Nature.

[11] Shah SP (2012). The clonal and mutational evolution spectrum of primary triple-negative breast cancers. Nature.

[12] Shah SP (2009). Mutational evolution in a lobular breast tumour profiled at single nucleotide resolution. Nature.

[13] Brock A, Chang H, Huang S (2009). Non-genetic heterogeneity—A mutation-independent driving force for the somatic evolution of tumours. Nat. Rev. Genet..

[14] Marusyk A, Janiszewska M, Polyak K (2020). Intratumor heterogeneity: The Rosetta stone of therapy resistance. Cancer Cell.

[15] Cortazar P (2014). Pathological complete response and long-term clinical benefit in breast cancer: The CTNeoBC pooled analysis. Lancet Lond. Engl..

[16] von Minckwitz G (2012). Definition and impact of pathologic complete response on prognosis after neoadjuvant chemotherapy in various intrinsic breast cancer subtypes. J. Clin. Oncol. Off. J. Am. Soc. Clin. Oncol..

[17] Symmans WF (2017). Long-term prognostic risk after neoadjuvant chemotherapy associated with residual cancer burden and breast cancer subtype. J. Clin. Oncol. Off. J. Am. Soc. Clin. Oncol..

[18] Untch M (2010). Neoadjuvant treatment with trastuzumab in HER2-positive breast cancer: Results from the GeparQuattro study. J. Clin. Oncol. Off. J. Am. Soc. Clin. Oncol..

[19] Pierga J-Y (2010). A multicenter randomized phase II study of sequential epirubicin/cyclophosphamide followed by docetaxel with or without celecoxib or trastuzumab according to HER2 status, as primary chemotherapy for localized invasive breast cancer patients. Breast Cancer Res. Treat..

[20] Risom T (2018). Differentiation-state plasticity is a targetable resistance mechanism in basal-like breast cancer. Nat. Commun..

[21] Rye IH (2018). Intratumor heterogeneity defines treatment-resistant HER 2+ breast tumors. Mol. Oncol..

[22] Bhang HC (2015). Studying clonal dynamics in response to cancer therapy using high-complexity barcoding. Nat. Med..

[23] Sharma A (2018). Longitudinal single-cell RNA sequencing of patient-derived primary cells reveals drug-induced infidelity in stem cell hierarchy. Nat. Commun..

[24] Yates LR (2015). Subclonal diversification of primary breast cancer revealed by multiregion sequencing. Nat. Med..

[25] Balko JM (2014). Molecular profiling of the residual disease of triple-negative breast cancers after neoadjuvant chemotherapy identifies actionable therapeutic targets. Cancer Discov..

[26] Almendro V (2014). Inference of tumor evolution during chemotherapy by computational modeling and in situ analysis of genetic and phenotypic cellular diversity. Cell Rep..

[27] Macosko EZ (2015). Highly parallel genome-wide expression profiling of individual cells using nanoliter droplets. Cell.

[28] Subramanian A (2005). Gene set enrichment analysis: A knowledge-based approach for interpreting genome-wide expression profiles. Proc. Natl. Acad. Sci..

[29] Webber JT, Kaushik S, Bandyopadhyay S (2018). Integration of tumor genomic data with cell lines using multi-dimensional network modules improves cancer pharmacogenomics. Cell Syst..

[30] Parker JS (2009). Supervised risk predictor of breast cancer based on intrinsic subtypes. J. Clin. Oncol..

[31] Dunbier AK (2011). Association between breast cancer subtypes and response to neoadjuvant anastrozole. Steroids.

[32] Liberzon A (2015). The molecular signatures database hallmark gene set collection. Cell Syst..

[33] Creedon H (2016). Identification of novel pathways linking epithelial-to-mesenchymal transition with resistance to HER2-targeted therapy. Oncotarget.

[34] Lesniak D (2013). Spontaneous epithelial-mesenchymal transition and resistance to HER-2-targeted therapies in HER-2-positive luminal breast cancer. PloS One.

[35] DeRose YS (2011). Tumor grafts derived from women with breast cancer authentically reflect tumor pathology, growth, metastasis and disease outcomes. Nat. Med..

[36] Liu H (2019). Tumor-derived IFN triggers chronic pathway agonism and sensitivity to ADAR loss. Nat. Med..

[37] Wolf DM, Lenburg ME, Yau C, Boudreau A, van’t Veer LJ (2014). Gene co-expression modules as clinically relevant hallmarks of breast cancer diversity. PLOS ONE.

[38] Network CGA (2012). Comprehensive molecular portraits of human breast tumours. Nature.

[39] Tickle T, Tirosh I, Georgescu C, Brown M, Haas B (2019). InferCNV of the Trinity CTAT Project.

[40] Borden EC (2019). Interferons α and β in cancer: Therapeutic opportunities from new insights. Nat. Rev. Drug Discov..

[41] Duijf PHG (2019). Mechanisms of genomic instability in breast cancer. Trends Mol. Med..

[42] Lee HJ (2016). Differential expression of major histocompatibility complex class I in subtypes of breast cancer is associated with estrogen receptor and interferon signaling. Oncotarget.

[43] Sporikova Z, Koudelakova V, Trojanec R, Hajduch M (2018). Genetic markers in triple-negative breast cancer. Clin. Breast Cancer.

[44] Mackenzie KJ (2017). cGAS surveillance of micronuclei links genome instability to innate immunity. Nature.

[45] Daemen A (2013). Modeling precision treatment of breast cancer. Genome Biol..

[46] Ramanjulu JM (2018). Design of amidobenzimidazole STING receptor agonists with systemic activity. Nature.

[47] Hafner M, Niepel M, Chung M, Sorger PK (2016). Growth rate inhibition metrics correct for confounders in measuring sensitivity to cancer drugs. Nat. Methods.

[48] Clark NA (2017). GRcalculator: An online tool for calculating and mining dose–response data. BMC Cancer.

[49] Pal B (2021). A single-cell RNA expression atlas of normal, preneoplastic and tumorigenic states in the human breast. EMBO J..

[50] Echeverria GV (2019). Resistance to neoadjuvant chemotherapy in triple-negative breast cancer mediated by a reversible drug-tolerant state. Sci. Transl. Med..

[51] Magbanua MJM (2015). Serial expression analysis of breast tumors during neoadjuvant chemotherapy reveals changes in cell cycle and immune pathways associated with recurrence and response. Breast Cancer Res. BCR.

[52] Puram SV (2017). Single-cell transcriptomic analysis of primary and metastatic tumor ecosystems in head and neck cancer. Cell.

[53] Filbin MG (2018). Developmental and oncogenic programs in H3K27M gliomas dissected by single-cell RNA-seq. Science.

[54] Tirosh I (2016). Dissecting the multicellular ecosystem of metastatic melanoma by single-cell RNA-seq. Science.

[55] Chung W (2017). Single-cell RNA-seq enables comprehensive tumour and immune cell profiling in primary breast cancer. Nat. Commun..

[56] Roden DL (2018). Single cell transcriptomics reveals molecular subtype and functional heterogeneity in models of breast cancer. bioRxiv.

[57] Boelens MC (2014). Exosome transfer from stromal to breast cancer cells regulates therapy resistance pathways. Cell.

[58] Gaston J (2016). Intracellular STING inactivation sensitizes breast cancer cells to genotoxic agents. Oncotarget.

[59] Weichselbaum RR (2008). An interferon-related gene signature for DNA damage resistance is a predictive marker for chemotherapy and radiation for breast cancer. Proc. Natl. Acad. Sci. U. S. A..

[60] Benci JL (2016). Tumor interferon signaling regulates a multigenic resistance program to immune checkpoint blockade. Cell.

[61] Bakhoum SF (2018). Chromosomal instability drives metastasis through a cytosolic DNA response. Nature.

[62] Neve RM (2006). A collection of breast cancer cell lines for the study of functionally distinct cancer subtypes. Cancer Cell.

[63] Yang X (2011). A public genome-scale lentiviral expression library of human ORFs. Nat. Methods.

[64] Camarda R (2016). Inhibition of fatty acid oxidation as a therapy for MYC-overexpressing triple-negative breast cancer. Nat. Med..

[65] Lawson DA (2015). Single-cell analysis reveals a stem-cell program in human metastatic breast cancer cells. Nature.

[66] Butler A, Hoffman P, Smibert P, Papalexi E, Satija R (2018). Integrating single-cell transcriptomic data across different conditions, technologies, and species. Nat. Biotechnol..

[67] Stuart T (2019). Comprehensive integration of single-cell data. Cell.

[68] Satija R, Farrell JA, Gennert D, Schier AF, Regev A (2015). Spatial reconstruction of single-cell gene expression data. Nat. Biotechnol..

[69] Yu G, Wang L-G, Han Y, He Q-Y (2012). clusterProfiler: An R package for comparing biological themes among gene clusters. OMICS J. Integr. Biol..

[70] Patel AP (2014). Single-cell RNA-seq highlights intratumoral heterogeneity in primary glioblastoma. Science.

[71] Schindelin J (2012). Fiji: An open-source platform for biological-image analysis. Nat. Methods.

