# Supplementary material for: An inflamed tumor cell subpopulation promotes chemotherapy resistance in triple negative breast cancer
Source: Sci Rep. 2024 Feb 14;14:3694. doi: 10.1038/s41598-024-53999-w (PMC10866903; doi:10.1038/s41598-024-53999-w)
Supplement: Supplementary file 6 — Supplementary Legends. [file 41598_2024_53999_MOESM6_ESM.docx]

**An inflamed tumor cell subpopulation promotes chemotherapy resistance in triple negative breast cancer**

**Mauricio Jacobo Jacobo,**^1,4^ **Hayley J. Donnella,**^1,4^ **Sushil Sobti**,^1^ **Swati Kaushik**,^1^ **Andrei Goga**,^2,3^ **Sourav** **Bandyopadhyay**^1,5,*^

^1^Department of Bioengineering and Therapeutic Sciences, University of California San Francisco, San Francisco, CA 94143, USA.

^2^Department of Cell & Tissue Biology, University of California San Francisco, San Francisco, CA 94143, USA.

^3^Department of Medicine, University of California San Francisco, San Francisco, CA 94143, USA

^4^These authors contributed equally

^5^Lead contact

^*^Correspondence: sourav.bandyopadhyay@ucsf.edu­ (S.B.)

**SUPPLEMENTARY DATASETS**

**Supplementary Dataset 1.** Single-cell RNA sequencing metrics summary.

**Supplementary Dataset 2.** Differentially expressed genes by cluster for each breast cancer cell line and patient-derived xenograft model sequenced and overrepresented gene sets in inflamed clusters.

**Supplementary Dataset 3.** Gene set enrichment analysis (GSEA) of ER+ MCF7 Cluster 4 cells and HER2+ SKBR3 Cluster 6 cells.

**Supplementary Dataset 4.** EMT, Basal, and Interferon-stimulated gene (ISG) module gene sets.

**Supplementary Dataset 5.** Gene set enrichment analysis (GSEA) of HLA^HI^ versus HLA^LO^ HCC38 and MDA-MB-468 cells
