# Supplementary material for: An inflamed tumor cell subpopulation promotes chemotherapy resistance in triple negative breast cancer
Source: Sci Rep. 2024 Feb 14;14:3694. doi: 10.1038/s41598-024-53999-w (PMC10866903; doi:10.1038/s41598-024-53999-w)
Supplement: Supplementary file 7 — Supplementary Figures. [file 41598_2024_53999_MOESM7_ESM.docx]

**An inflamed tumor cell subpopulation promotes chemotherapy resistance in triple negative breast cancer**

**Mauricio Jacobo Jacobo,**^1,4^ **Hayley J. Donnella,**^1,4^ **Sushil Sobti**,^1^ **Swati Kaushik**,^1^ **Andrei Goga**,^2,3^ **Sourav** **Bandyopadhyay**^1,5,*^

^1^Department of Bioengineering and Therapeutic Sciences, University of California San Francisco, San Francisco, CA 94143, USA.

^2^Department of Cell & Tissue Biology, University of California San Francisco, San Francisco, CA 94143, USA.

^3^Department of Medicine, University of California San Francisco, San Francisco, CA 94143, USA

^4^These authors contributed equally

^5^Lead contact

^*^Correspondence: sourav.bandyopadhyay@ucsf.edu­ (S.B.)

**SUPPLEMENTAL INFORMATION**

**SUPPLEMENTAL FIGURES AND LEGENDS**


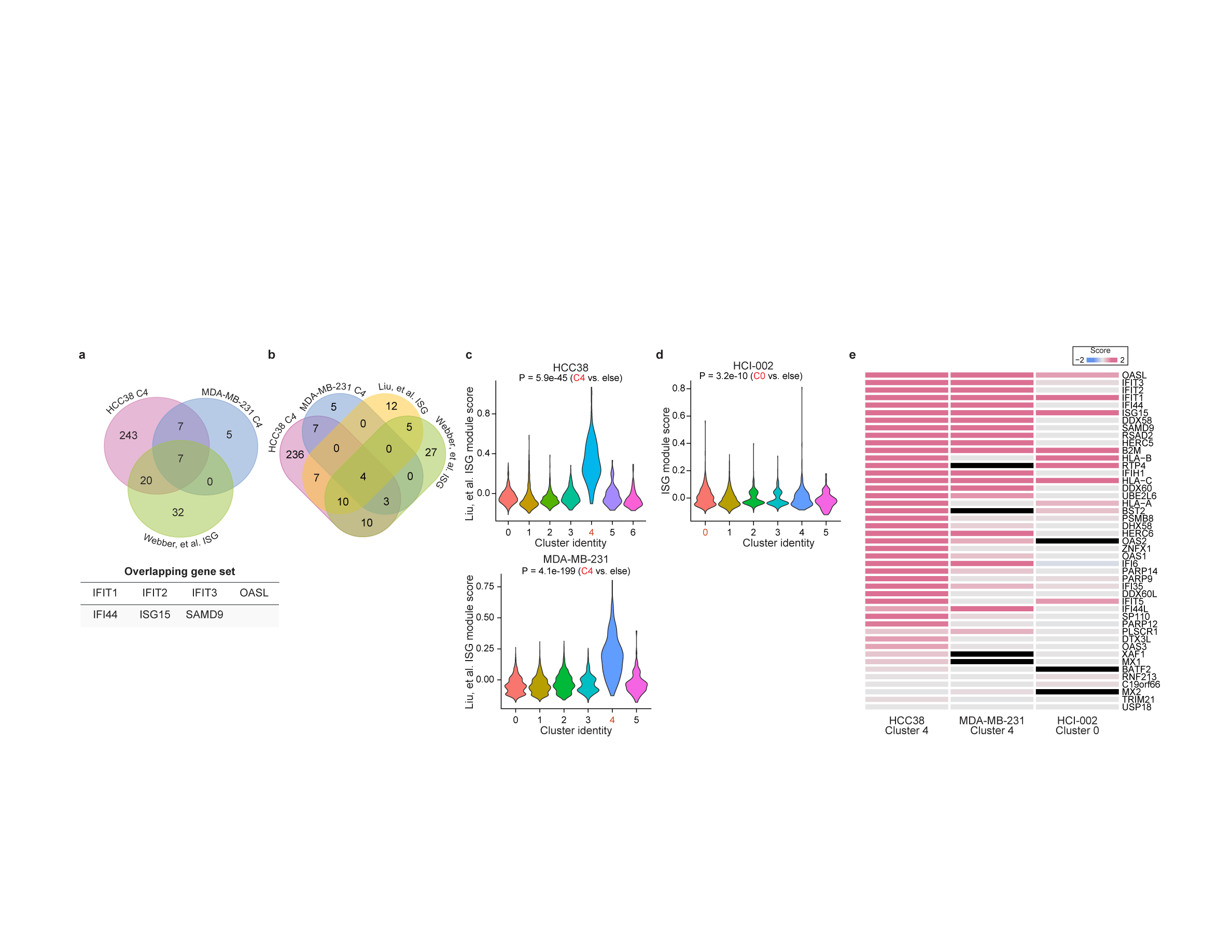


**Supplementary Figure 1. ISG signature overlap in TNBC cells. a**, Overlap of genes in the ISG module identified in Webber, et al.^S1^, and differentially expressed genes from HCC38 Cluster 4 and MDA-MB-231 Cluster 4 cells. Genes overlapping between all three gene sets are shown below. **b**, Overlap of genes identified in published ISG signatures (Webber, et al. and Liu, et al.)^S1,S2^ in comparison to differentially expressed gene lists from HCC38 Cluster 4 and MDA-MB-231 Cluster 4 cells. **c**, Relative ISG module scores using the Liu, et al.^S2^ ISG signature shown for individual HCC38 (top) and MDA-MB-231 cells (bottom). **d**, Relative ISG module scores for individual HCI-002 cells in each cluster. **e**, Relative scores of ISG module genes and MHC-I genes in HCC38 Cluster 4 cells, MDA-MB-231 Cluster 4 cells, and HCI-002 Cluster 0 cells compared to all other cells from the respective dataset. A black bar indicates an absent value. In all graphs *P* values are calculated using a two-sided Wilcoxon test as indicated.

**
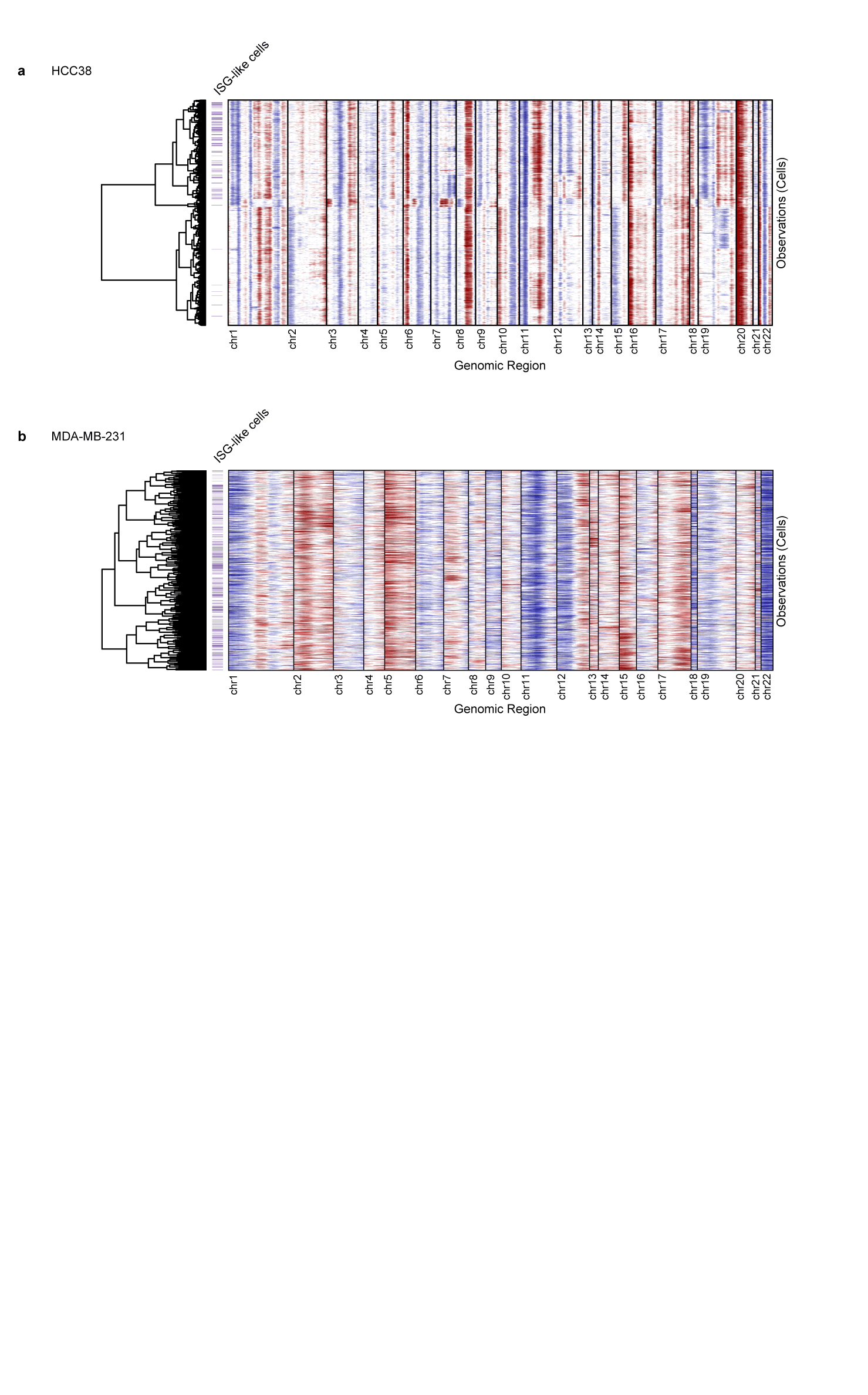
**

**Supplementary Figure 2. Genetic factors do not contribute to the presence of inflamed cells in TNBC in vitro models.**  Hierarchical clustering of inferred CNV profiles of individual (**a**) HCC38 and (**b**) MDA-MB-231 cells. Chromosomal regions are shown with amplifications (red) or deletions (blue). Transcriptional identification of ISG-enriched, inflamed cells is also displayed (purple).


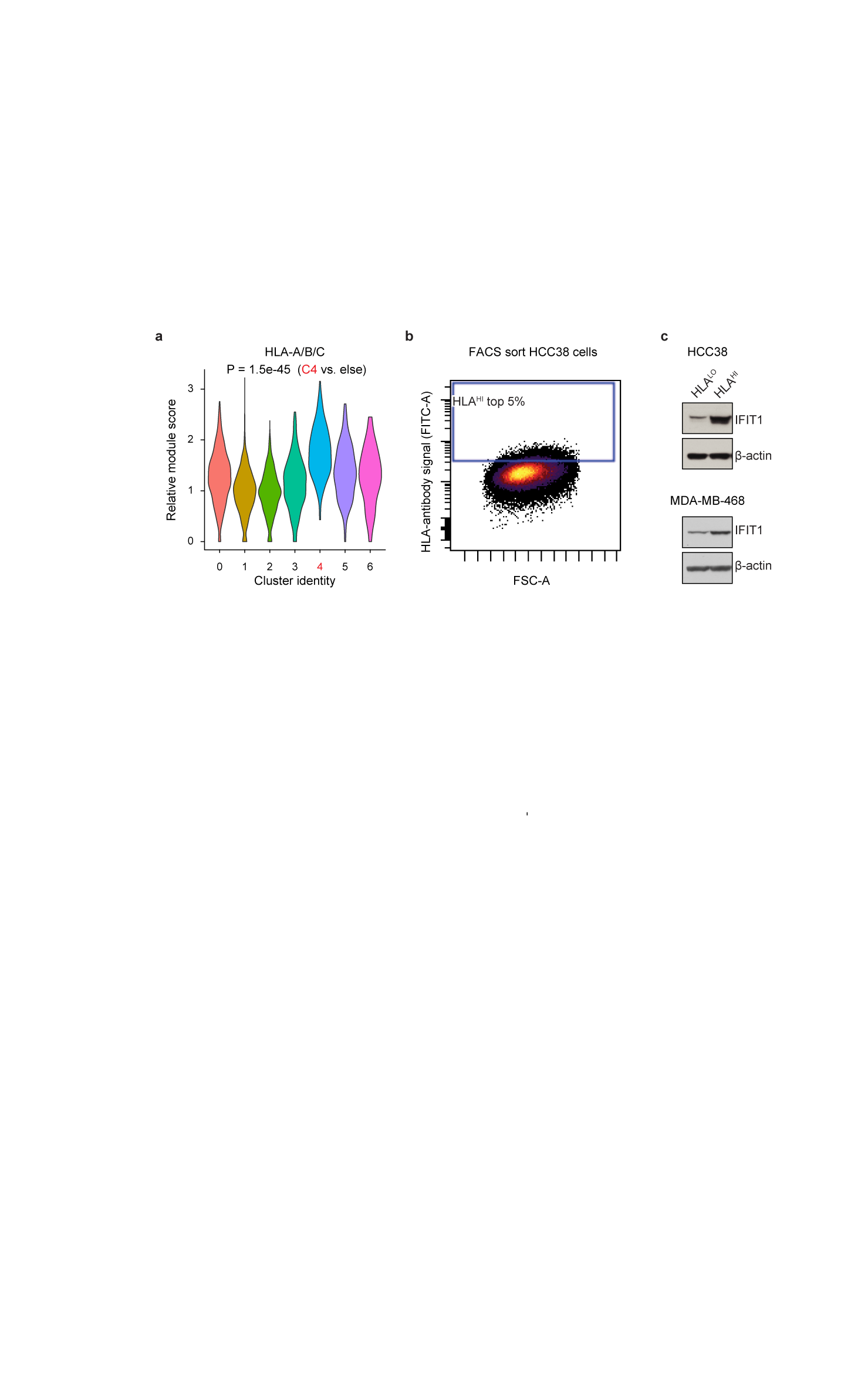


**Supplementary Figure 3. Inflamed cells characterized by heightened expression of MHC class I molecules.** **a**, HCC38 cells were scored based on their combined expression of *HLA-A*, *HLA-B*, and *HLA-C*. Violin plot of the relative gene module score for individual cells within each cluster is shown. *P* value calculated using a two-sided Wilcoxon test. **b**, Inflamed cells were isolated by sorting HCC38 HLA^HI^ (top 5%) cell fractions based on a pan-HLA-A/B/C antibody signal. **c**, Protein lysates of HCC38 (top) and MDA-MB-468 (bottom) HLA^LO^ (bottom 10%) and HLA^HI^ (top 5%) fractions subjected to immunoblot analysis using the indicated antibodies. Representative image from *n* = 3 independent experiments.

**
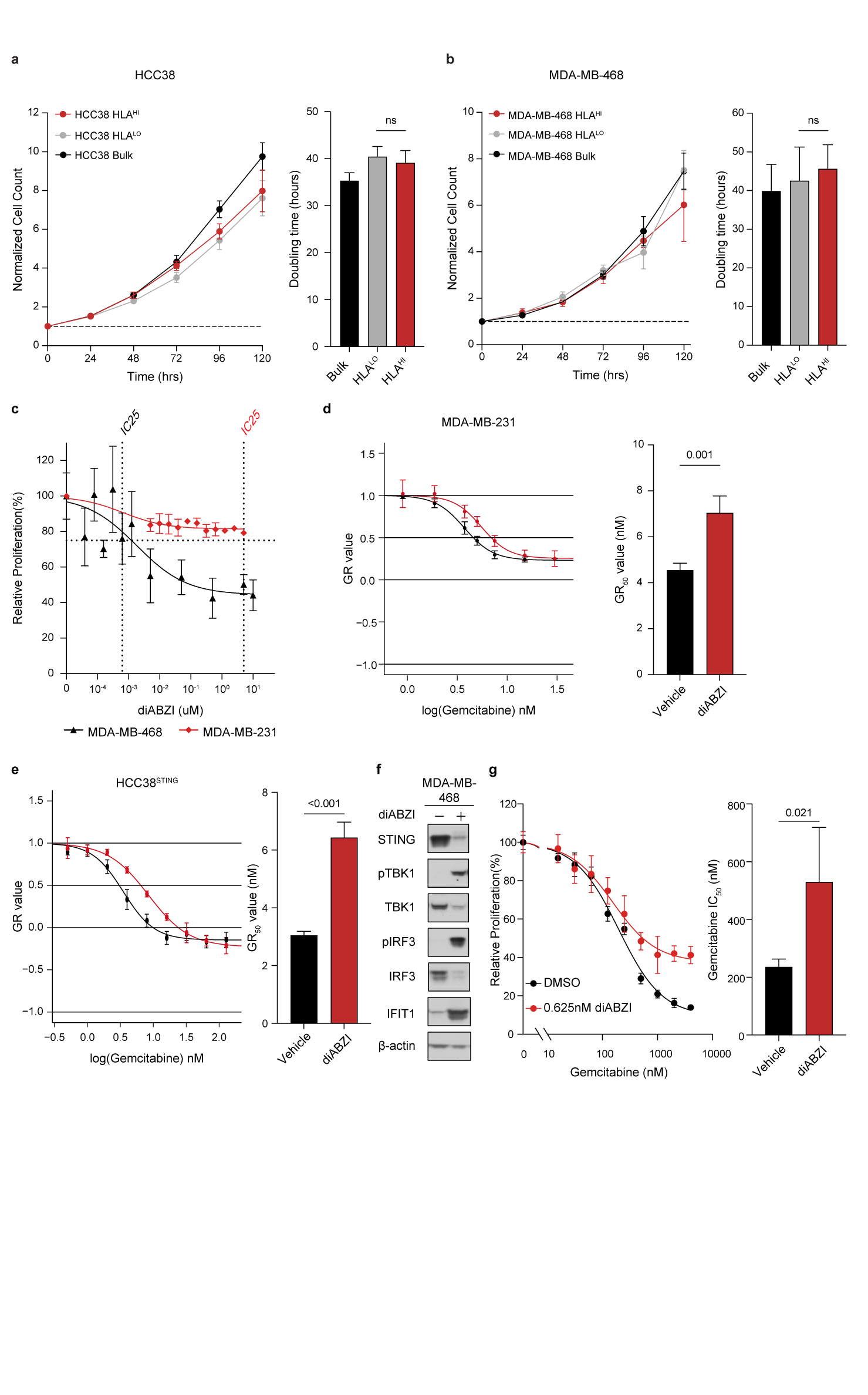
**

**Supplementary Figure 4. FACS sorted and diABZI treated TNBC cells have no to minimal effect on the growth rate of cells. a**, Proliferation of HCC38 HLA sorted cells over 5 d normalized to day 0. Dashed line represents the day 0 normalized baseline measurement. Average doubling time of HCC38 HLA populations expressed in hours shown to the right. **b**, Proliferation of MDA-MB-468 HLA sorted cells over 5 d normalized to day 0. Dashed line represents the day 0 normalized baseline measurement. Average doubling time of MDA-MB-468 HLA subpopulations expressed in hours shown to the right. **c**, Proliferation of MDA-MB-468 and MDA-MB-231 cells in response to 72 h diABZI treatment. Indicated concentrations correspond to the IC_25_ dose. **d**, **e** Growth rate inhibition measurements^S3,S4^ of (**d**) MDA-MB-231 cells (**e**) HCC38^STING^ cells in response to 24 h diABZI or DMSO pre-treatment followed by 72 h gemcitabine co-treatment. GR_50_ quantification of dose-response curves are shown to the right. **f**, Immunoblot of lysates taken after 24 hours of 5uM diABZI or DMSO treatment in MDA-MB-468 cells with the indicated antibodies. β-actin is shown as a loading control. Representative image from *n* = 3 independent experiments. **e**, Proliferation of MDA-MB-468 cells in response to 24 h diABZI or DMSO pre-treatment followed by 72 h gemcitabine co-treatment. IC_50_ quantification of MDA-MB-468 dose-response curve shown to the right. For (**a**,**b)** data represents *n* = 8 biologically independent samples and error bars are mean ± s.d. For (**c**, **d**, **e**, **g**) data represents *n* = 4 biologically independent samples and error bars are mean ± s.d. *P* value was calculated using a two-sided *t*-test. n.s. = not significant by a *t*-test.


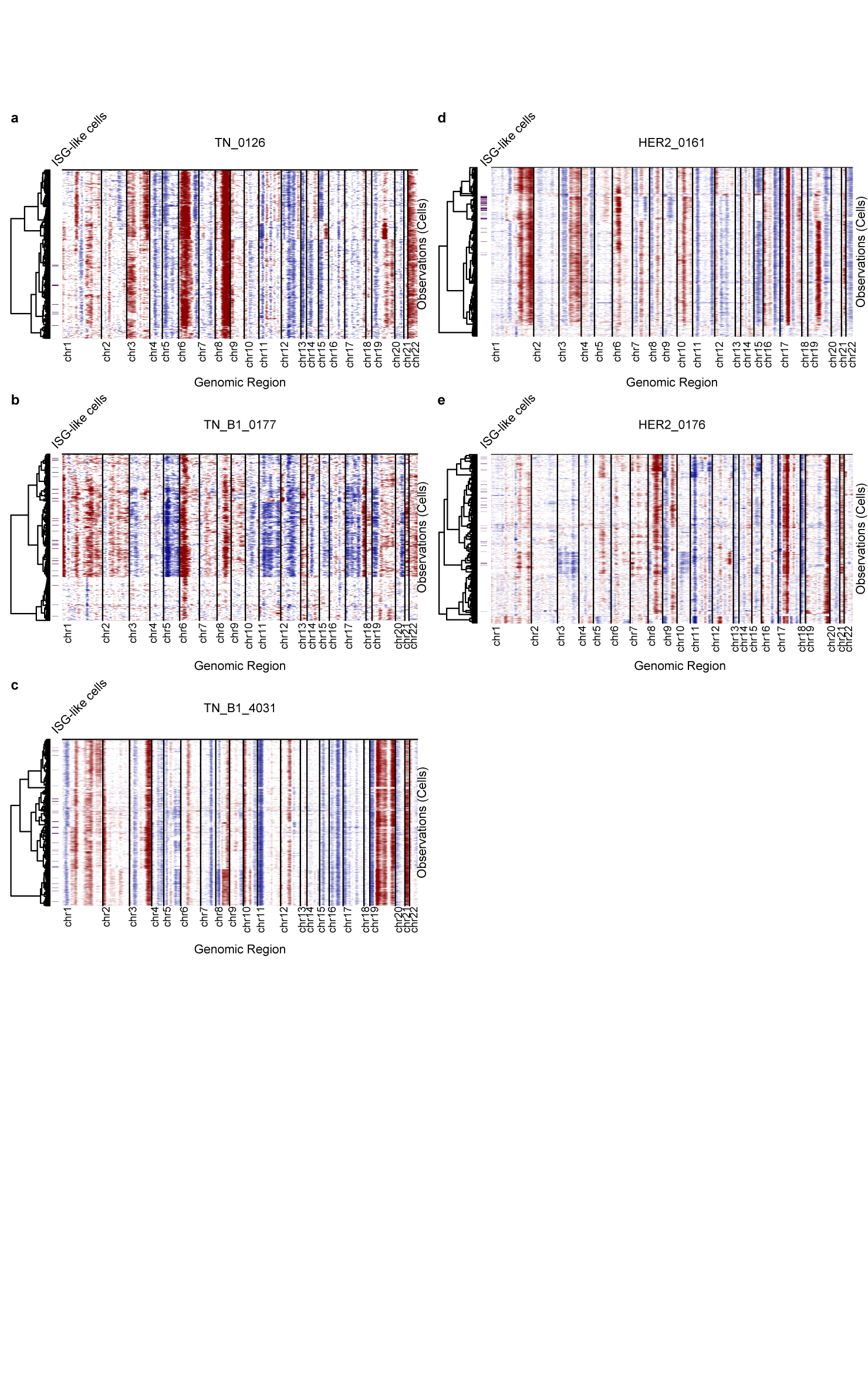


**Supplementary Figure 5. Genetic factors do not contribute to the presence of inflamed cells in TNBC or HER2+ clinical samples.** Hierarchical clustering of inferred CNV profiles of individual cells from (**a**) TN_0126, (**b**) TN_B1_0177, (**c**) TN_B1_4031, (**d**) HER2_0161, (**e**) HER2_0176 tumors. Chromosomal regions are shown with amplifications (red) or deletions (blue). Transcriptional identification of ISG-enriched, inflamed cells is also displayed for each patient (purple).


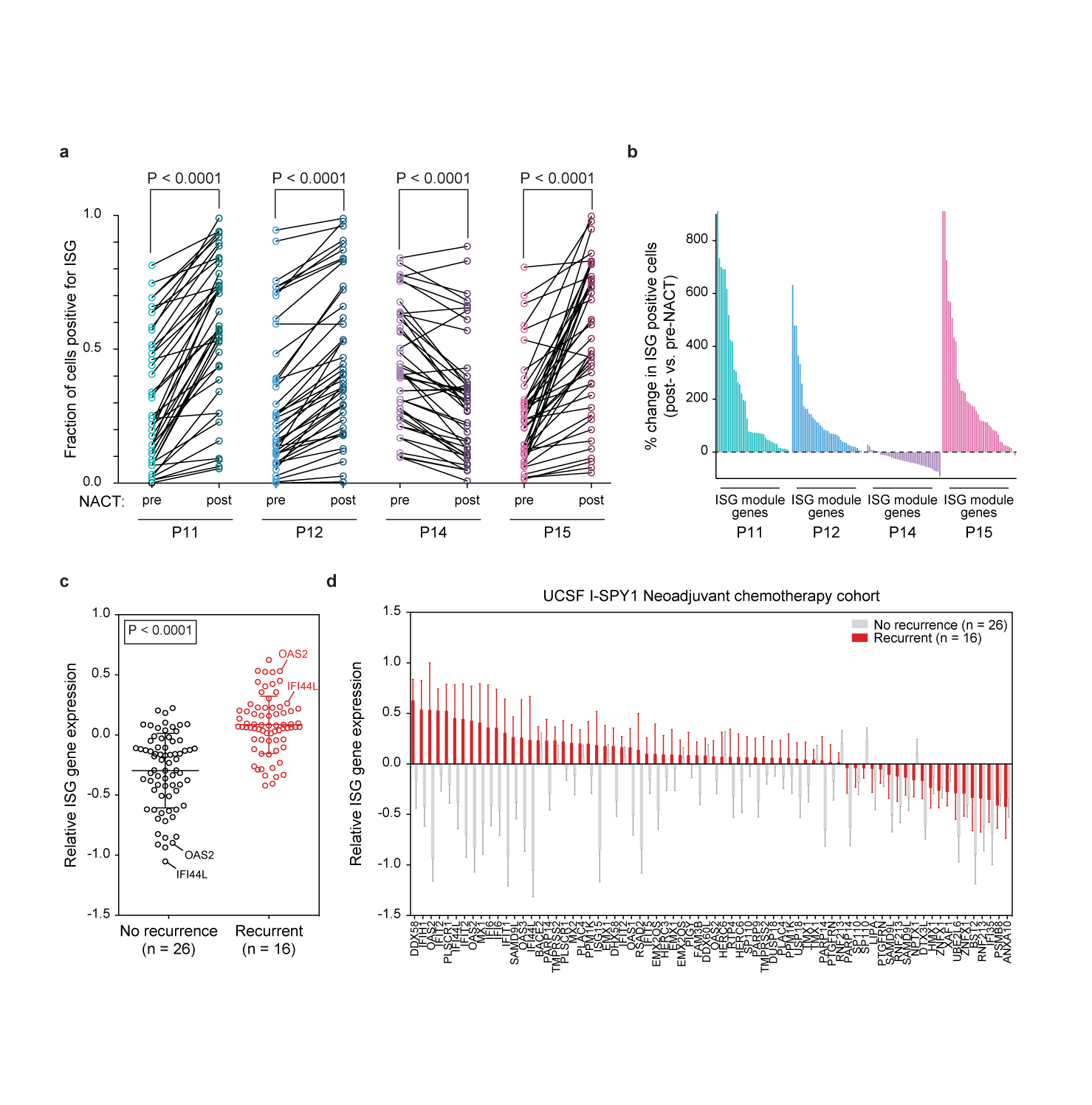


**Supplementary Figure 6. ISG enrichment in residual tumors from post-neoadjuvant chemotherapy (NACT) breast tumor samples. a**, For each ISG module gene, the fraction of single cells expressing it (>0 TPM) is shown between matched between pre- and post-NACT biopsies from the same TNBC patient. **b**, Percent change in number of cells expressing ISG module genes after NACT from 4 TNBC patients. **c**, Relative expression of ISG module genes in residual compared to pre-treatment tumors that recurred (*n* = 16) during the I-SPY1 study^S5^ compared to samples from patients with no recurrence (*n* = 26). Each point represents one gene whose relative expression is averaged over a set of tumors, with OAS2 and IFI44L indicated for clarity. **d**, Expression of individual ISG module genes in residual tumors normalized to pre-treatment from patients that recurred or not in the I-SPY1 trial. Error bars are mean ± s.d. *P* values calculated using paired *t*-test.


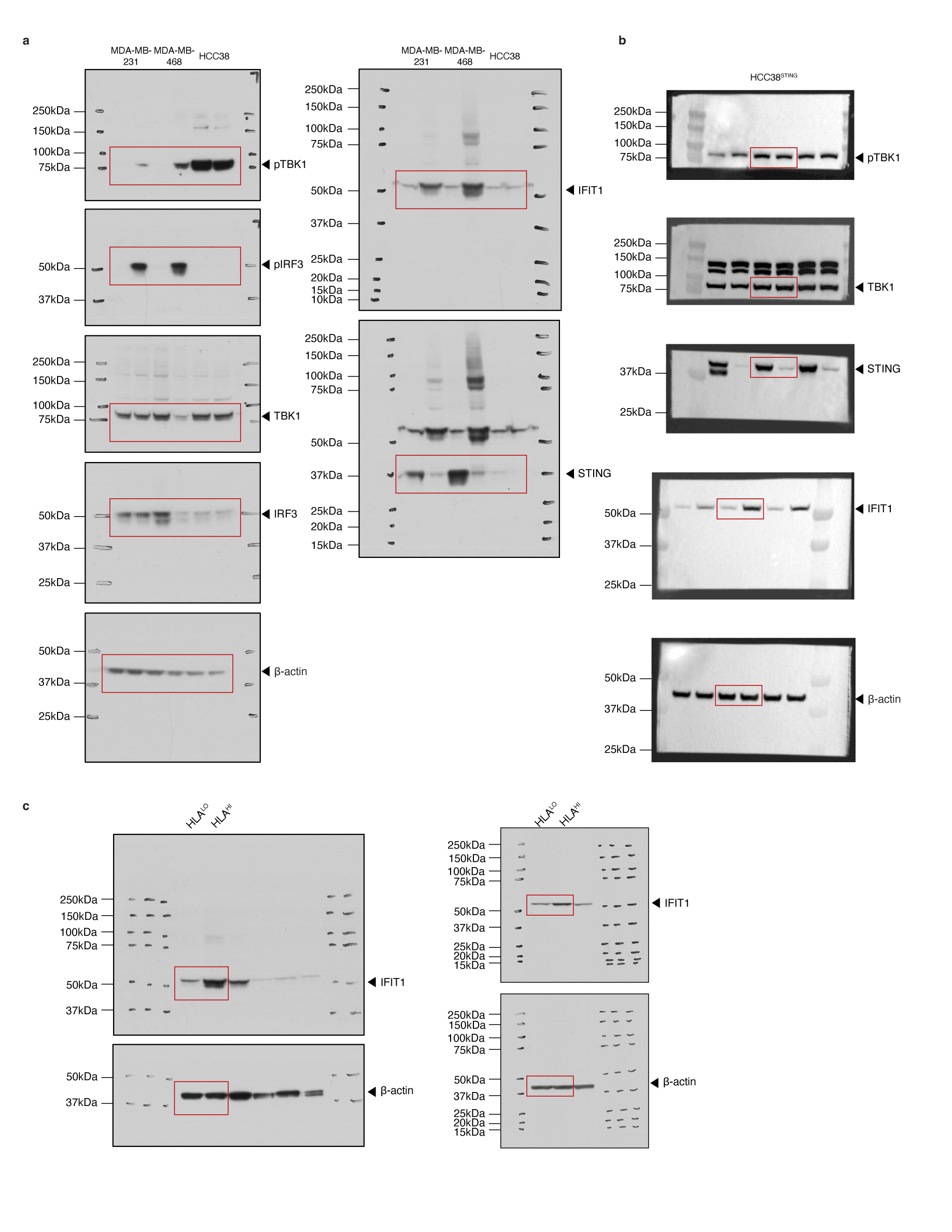


**Supplementary Figure 7. Full blot images.** Regions surrounded by red boxes are presented in (**a**) Fig. 5a, 5d, and Supplementary Fig. 4f, (**b**) Fig.5d, and (**c**) Supplementary Fig. 3c.

**SUPPLEMENTAL REFERENCES**

S1. Webber, J. T., Kaushik, S. & Bandyopadhyay, S. Integration of Tumor Genomic Data with Cell Lines Using

Multi-dimensional Network Modules Improves Cancer Pharmacogenomics. *Cell Syst* **7**, 526-536.e6 (2018).

S2. Liu, H. *et al.* Tumor-derived IFN triggers chronic pathway agonism and sensitivity to ADAR loss. *Nat Med*

**25**, 95–102 (2019).

S3. Hafner, M., Niepel, M., Chung, M. & Sorger, P. K. Growth rate inhibition metrics correct for confounders in

measuring sensitivity to cancer drugs. *Nat Methods* **13**, 521–527 (2016).

S4. Clark, N. A. *et al.* GRcalculator: an online tool for calculating and mining dose–response data. *BMC*

*Cancer* **17**, 698 (2017).

S5. Magbanua, M. J. M. *et al.* Serial expression analysis of breast tumors during neoadjuvant chemotherapy

reveals changes in cell cycle and immune pathways associated with recurrence and response. *Breast*

*Cancer Res* **17**, 73 (2015).
